# Supplementary material for: Stability of ecologically scaffolded traits during evolutionary transitions in individuality
Source: Nat Commun. 2024 Aug 3;15:6566. doi: 10.1038/s41467-024-50625-1 (PMC11297203; doi:10.1038/s41467-024-50625-1)
Supplement: Supplementary file 1 — Supplementary information [file 41467_2024_50625_MOESM1_ESM.pdf]

Supplementary information for: “Stability of Ecologically Scaffolded Traits During Evolutionary Transitions in Individuality”

Guilhem Doulcier *[1,2], Peter Takacs [4,1], Katrin Hammerschmidt †[3], and Pierrick Bourrat †[1,4,5]

[1]Philosophy Department, Macquarie University, New South Wales 2109, Australia

[4]Department of Philosophy and Charles Perkins Centre, The University of Sydney, New South Wales 2006, Australia

[5]ARC Centre of Excellence in Synthetic Biology, Sydney, Australia

[3]Institute of Microbiology, Kiel University, Kiel, Germany

[2]Max Planck Institute for Evolutionary Biology, Plön, Germany

June 28, 2024

Contents

1 Stochastic Simulations **2**

2 Eco-Evolutionary Meanfield Patch Model **5**

3 Stochastic Patch Model **8**

4 Conditions for Endogenisation **11**

5 Selection for Collective Size **13**

*Corresponding authors: guilhem.doulcier@normalesup.org, katrinhammerschmidt@googlemail.com, p.bourrat@gmail.com

†These authors contributed equally

Supplementary Note 1 Stochastic Simulations

In this section, we describe the algorithm used for the stochastic simulations of the meta-population eco-evolutionary dynamics.

A simulation of the model takes as input:

- A *scaffold*, composed of an undirected graph $\mathcal{S} = (\mathcal{V}, \mathcal{E})$ where each vertex is a patch, and a function $\mathcal{R}$ that associates each patch to a number of resources. An edge between two vertices means that propagules from one patch can reach the other patch.
- A *trait space* $\mathcal{T}$, which encodes the possible trait values, and a mutation rate μ. Particles inherit the trait value of their parent with probability $(1-\mu)$ or mutate to an adjacent value in the trait space with probability μ.
- A *particle ecology* function that associates the trait value of a focal particle $\theta \in \mathcal{T}$, the number of available resources $R \in \mathbb{N}$, and the number of other particles in the patch $N \in \mathbb{N}$ to the probability that a particle would duplicate or form a propagule. $p : \theta, R, N \mapsto p(\theta, R, N)$
- An *initial population* of particles: that is, a number of particles with associated trait value and patch location.
- A *duration* of the simulation $T \in \mathbb{R}_+$.

We use the exact stochastic simulation algorithm (SSA) to generate the trajectory of the system, starting from time $t = 0$, and while $t < T$ (the time to the next event) is sampled from an exponential distribution, and the nature of the next event is sampled from a multinomial distribution with appropriate parameters.

The possible events are, for each particle:

- Particle birth, with rate p for each particle. Add a particle to the same patch, reduce the number of resources in the patch by 1. With probability μ, the new particle mutates to a different trait value than its parent.
- Propagule production, with rate $1-p$. Remove the particle, reduce the number of resources in the patch by 1. Select uniformly at random a patch adjacent to the current patch. If this patch does not contain any particles, add a new particle with the same trait value to this patch.

If after applying the event the number of resources in the focal patch e is 0, reset the patch: remove all particles and replenish the resources to its initial value $\mathcal{R}(e)$.

- In the intrinsic particle dispersal-duplication ratio, the ecology function is simply the constant function $p : \theta, R, N \mapsto \theta$.
- In the density-dependent particle dispersal-duplication ratio, the ecology function is:

$$p(\theta, R, N) = \begin{cases} \theta & \text{if } N = 1, \\ 1-\theta & \text{otherwise.} \end{cases} \quad (1)$$

Evolutionary trajectories

We now check that the meanfield approximation described in Section 2 is coherent with the stochastic simulations.

We start with a complete network (i.e., each patch is connected to each patch) and constant resource richness.

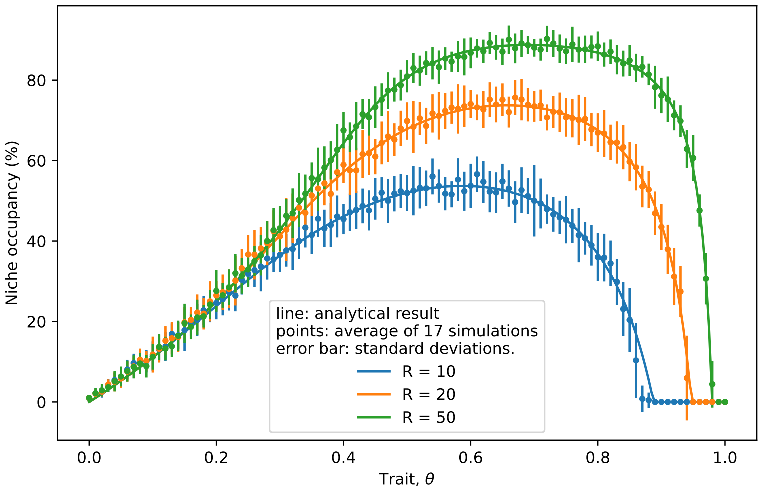

Supplementary Figure 1: **Occupancy with constant scaffolding conditions.** Proportion of patches that are occupied as a function of θ in the intrinsic duplication-dispersal ratio model. The scaffold is a complete graph of $D = 100$ patches with a uniform number R of resources, and no mutations are allowed. The line is the prediction of the meanfield model. Each dot is the average occupancy in the stochastic simulation (number of replicates in the legend). Error bars are the standard deviation to the mean.

Supplementary Figure 1 shows the occupancy of patches at steady state in the stochastic simulation (points). Note that the stochastic occupancy corresponds to the prediction of the meanfield model (line).

Supplementary Figure 2 shows the life span and number of propagules sent during the life span of the patch. Note that the expected value computed in Supplementary Section 3 is a good approximation of the results of the stochastic trajectories.

Supplementary Figure 3 shows the evolutionary trajectory obtained by stochastic simulations. Note that the endpoint of the simulations corresponds to the prediction of the meanfield model.

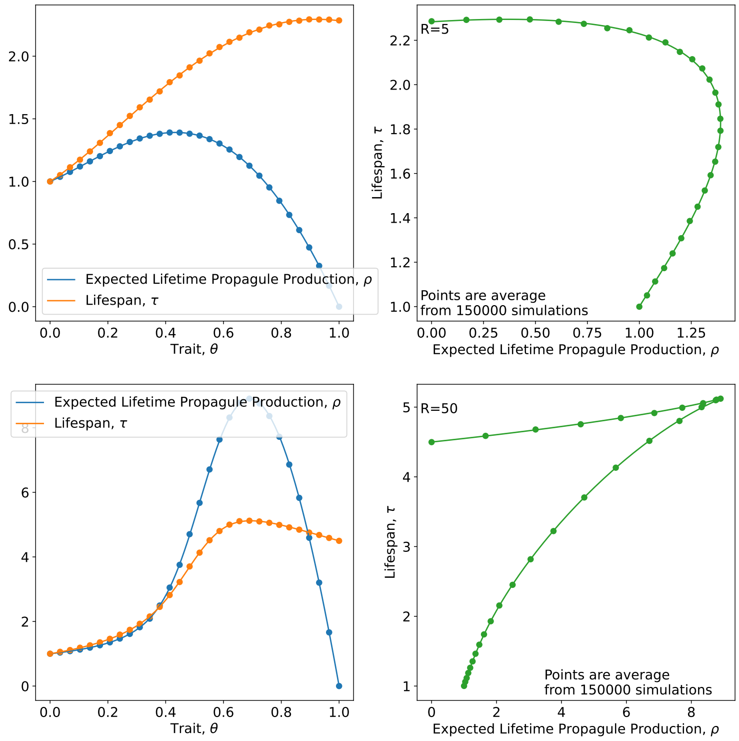

Supplementary Figure 2: **Single collective simulations** Only a single collective is simulated, with a number R of resources and a trait value θ in $[0, 1]$ for the intrinsic ratio.

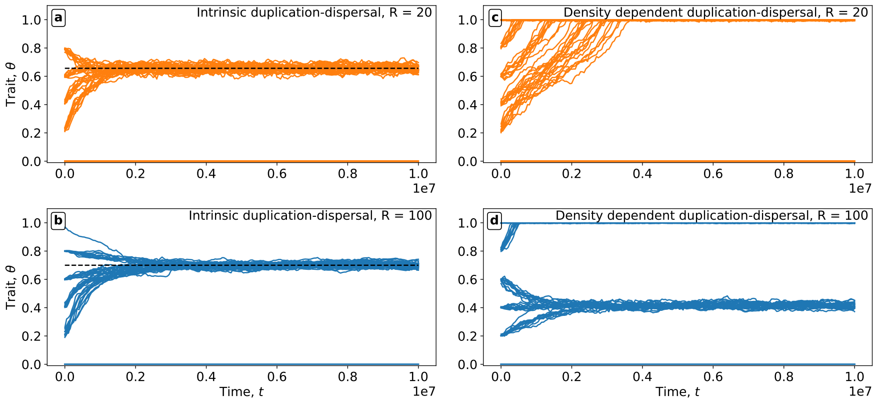

Supplementary Figure 3: **Evolutionary strategies with constant scaffolding conditions.** In these simulations, the scaffold is a complete graph of $D = 100$ patches with a uniform number R of resources and an initial trait value θ_0 in $(0, 0.2, ...0.8, 1)$ for both ratio functional forms. Each line is an independent stochastic simulation, and 10 replicates are simulated for each set of conditions. The dotted line corresponds to the adaptive dynamics prediction in the meanfield model.

Supplementary Note 2 Eco-Evolutionary Meanfield Patch Model

In this section, we study the meanfield model approximating the eco-evolutionary dynamics of the meta-population (a textbook description of this kind of approximation can be found in [1]). We establish the expression of the invasion fitness gradient and study the associated pairwise invasibility plot.

A Description

Let $C(t)$ be the density of occupied patches at time t and $E(t)$ be the density of empty patches at time t. Consider that C and E follow Susceptible-Infected-Susceptible (SIS) dynamics:

$$\begin{cases} \frac{dC}{dt} = CE\frac{\rho}{\tau} - C\frac{1}{\tau} \\ \frac{dE}{dt} = -CE\frac{\rho}{\tau} + C\frac{1}{\tau} \end{cases} . \tag{2}$$

In this equation, ρ is the expected number of propagules sent by an occupied patch, and τ is the expected life span of a population within a patch. These values are derived from the stochastic model, as described in supplementary note 3. The initial conditions are (C_0, E_0), with $C_0 > 0$ and $E_0 + C_0 = 1$. In the following, the patch occupancy is the value of C expressed as a percentage.

B Ecological equilibrium

The ecological equilibrium is reached when $\frac{dC}{dt} = \frac{dE}{dt} = 0$. There are two branches of equilibria:

$$\begin{cases} C^* = 0 & \text{stable if } \rho < 1, \text{ (extinction)}, \\ C^* = 1 - \frac{1}{\rho} & \text{stable if } \rho > 1 \text{ (endemic)} \end{cases} \tag{3}$$

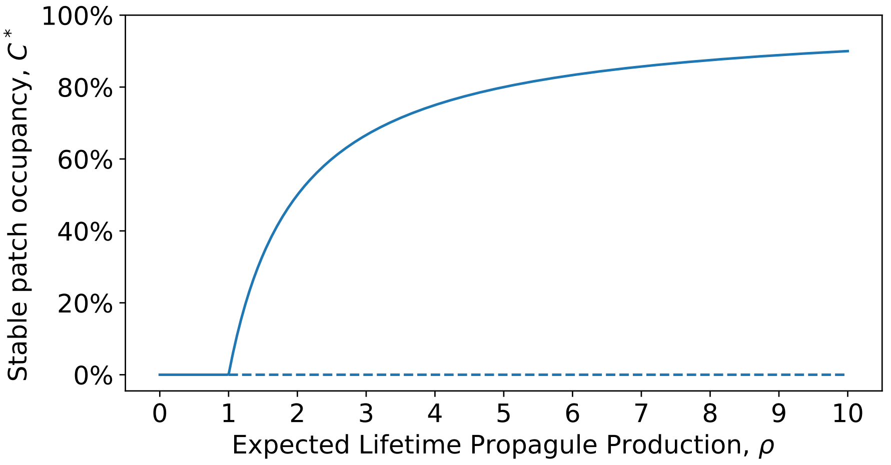

Supplementary Figure 4: Patch occupancy at equilibrium C^* as a function of the average propagule count.

In both cases, $E^* = D - C^*$. Note that there is a transcritical bifurcation in $\rho = 1$ (Supplementary Figure 4).

C Adaptive dynamics

Let $\tilde{\tau}$ and $\tilde{\rho}$ be functions of an underlying trait θ, such that $\tilde{\tau}(\theta) = \mathbb{E}(\tau|\theta)$ and $\tilde{\rho}(\theta) = \mathbb{E}(\rho|\theta)$.

First, we compute the invasion fitness from the ODE model by adding a mutant with a trait m in a population with resident trait r:

$$\begin{cases} \frac{dC}{dt}\tilde{\tau}(r) & = CE\tilde{\rho}(r) - C \\ \frac{dM}{dt}\tilde{\tau}(m) & = ME\tilde{\rho}(m) - M \\ \frac{dE}{dt} & = -ME\frac{\tilde{\rho}(m)}{\tilde{\tau}(m)} - CE\frac{\tilde{\rho}(r)}{\tilde{\tau}(r)} + C\frac{1}{\tau(r)} + M\frac{1}{\tau(m)} \end{cases} \tag{4}$$

Further, looking for the exponential growth rate of a rare mutant in a resident population at non-extinct equilibrium—that is, $E = \tilde{\rho}(r)^{-1}$ and C $= 1 - \tilde{\rho}(r)^{-1}$:

$$f(r, m) = \frac{1}{M}\frac{dM}{dt} = \frac{1}{\tilde{\tau}(m)}\left[\frac{\tilde{\rho}(m)}{\tilde{\rho}(r)} - 1\right] \tag{5}$$

Note that the model can be complexified by introducing a constant patch renewal flow instead of considering that the patch population is constant. If this model has a slightly different ecological equilibrium, the expression of the invasion fitness is the same. We do not reproduce this computation here in the interest of space, but the reader can verify this by replacing the term $+C\frac{1}{\tau}$ in Equation 2 with $+k$ with $k \in \mathbb{R}_+^*$.

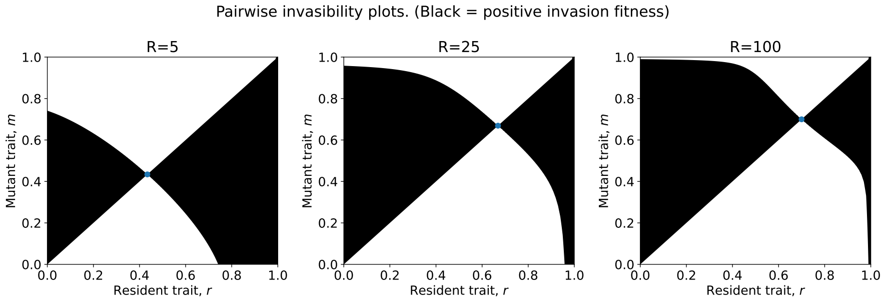

Supplementary Figure 5: **Pairwise invasibility plots for the intrinsic constant duplication-dispersal ratio.** In black are values of resident trait $\theta = r$ and mutant trait $\theta = m$ that correspond to a positive invasion fitness $f(r, m)$ and thus invasion by the mutant; in white are values that correspond to a negative invasion fitness and thus no invasion by the mutant. Invasions by a mutant with an infinitesimal trait difference are studied by looking at the sign changes along the first diagonal. A singular value (blue dot) occurs where the sign changes. In the three panels presented, it is always convergent and evolutionarily stable (see [2] for more details about the method of pairwise invasibility plots). R is the quantity of resources in a patch.

Supplementary Figure 5 shows the sign of the invasion fitness f as a function of θ for three values of R, taking the simplest functional form for p as a function of the trait θ, taking $p(\theta) = \theta$ (referred to as the intrinsic constant duplication-dispersal ratio in the main text).

The invasion fitness gradient g (i.e., the derivative of the invasion fitness evaluated when the mutant trait is equal to the resident trait) is:

$$g(\theta) = \frac{\partial f(\theta, m)}{\partial m}\Big|_{m=\theta} = \frac{\tilde{\rho}'(\theta)}{\tilde{\rho}(\theta)\tilde{\tau}(\theta)} \tag{6}$$

In the approximation we are making (i.e., rare mutant, resident at ecological equilibrium), the life span τ does not matter for the endpoint of the evolutionary trajectory outside the lifetime reproductive output ρ. The ESS is simply the trait value that optimises ρ. This can be seen in the expression of g. Since τ and ρ are always strictly positive, the values of θ for which g is null are the values of θ for which ρ' is null, and hence the values of θ for which ρ is maximal (or minimal).

Supplementary Figure 6 shows the singular trait value in the intrinsic constant duplication-dispersal ratio model obtained by numerically computing the root of the function $g(\theta)$ or, equiv-

alently, the maximum of the function $\rho(\theta)$. (For the ESS in the density-dependent duplication-dispersal model, see Supplementary Figure 7).

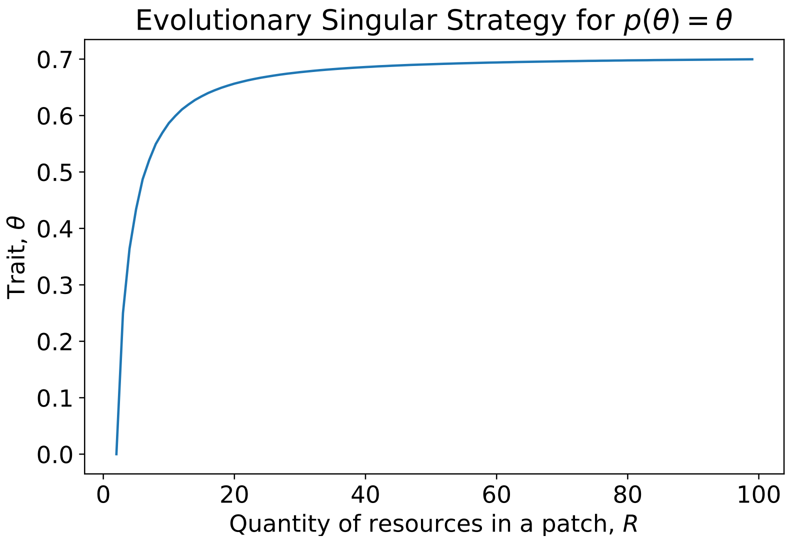

Supplementary Figure 6: **Evolutionarily singular strategies for the intrinsic constant duplication-dispersal ratio.**

One simplification of the modelling work we present is rooted in the adaptive dynamics approach, which supposes a separation of timescale between the introduction of new mutations and their fixation in the population. This limits the ability of this kind of model to quantitatively predict the duration T that would be required for scaffolding experiments to be successful. However, provided the fitness landscape has the same topology, qualitative results should hold.

Supplementary Note 3 Stochastic Patch Model

In this section, we study the stochastic model of particle dynamics within a patch and derive the expression for the expected life span and the expected number of propagules.

A Constant probability p

Let $(X_t, t \geq 0)$ be a one-dimensional continuous time Markov branching process modelling the number of particles in a patch. Let R be the number of resources in a patch, v be a timescale factor (set to 1 in the main text for easier presentation), and p be the probability to stay in the patch on a splitting event or send out a single propagule.

$(X_t, t \geq 0)$ is a linear birth-death process with per capita birth rate vp and per capita death rate $v(1-p)$. The process is stopped once all particles are dead ($X_t = 0$) or at the R-th splitting event (i.e., when the resources are depleted).

We are looking for the life span of the collective τ and the number of propagules ρ.

A.1 Expected values

The branching process $(X_t, t \geq 0)$ can be separated into two sub-processes: a jump process $(S_n, n = 1 \dots R)$ and a split time process $(T_n, n = 1 \dots R)$ [3, p. 118]. S_n is the number of cells at the n-th split time, and T_n is the time of the n-th split.

Jump process The jump process $(S_n, n = 1, 2 ... R)$ is a discrete time random walk, starting at $S_1 = 1$ and stopped if it reaches 0, with transition probabilities for $i > 0$ given by:

$$\mathbb{P}(S_n = i + 1 | S_{n-1} = i) = p \tag{7}$$

$$\mathbb{P}(S_n = i - 1 | S_{n-1} = i) = 1 - p := q \tag{8}$$

Consider a jump n such that $1 < n < R$—let us compute the discrete probability distribution $P(S_n = k)$ for all values of $0 < k < R$.

First case, $k \geq 1$ A trajectory of S is a path from the initial conditions $S_1 = 1$ to the final conditions, $S_k = n$ for which S is never 0. We can see this as points in the plane and note the state of the process as pairs of coordinates. Thus, we are looking for a path from $(1, 1)$ to (k, n) that never crosses the line $(\cdot, 0)$.

- It can be proven by reflection that there are $\frac{k}{n}\binom{n}{\frac{n+k}{2}}$ of such paths (through Bertrand's Ballot theorem).
- A path from $(1, 1)$ to (k, n) is composed of $(n+k)/2 - 1$ steps up and $(n-k)/2$ steps down. The probability of any such path is $p^{\frac{n+k}{2}-1}q^{\frac{n-k}{2}}$.

The probability $\mathbb{P}(S_n = k)$ is the product of this probability of getting any path from $(1, 1)$ to (k, n), with the number of such path. Thus:

$$\mathbb{P}(S_n = k) = \frac{k}{n}\binom{n}{\frac{n+k}{2}} p^{\frac{n+k}{2}-1} q^{\frac{n-k}{2}} \tag{9}$$

Second case, $k = 0$

$$\mathbb{P}(S_n = 0) = q\mathbb{P}(S_{n-1} = 1) = \frac{1}{n-1}\binom{n}{\frac{n}{2}} p^{\frac{n}{2}-1} q^{\frac{n}{2}} \tag{10}$$

Number of propagules There are as many propagules produced for a given trajectory of S as there are steps down in the corresponding path. Thus:

$$\mathbb{E}(\rho) = \frac{1}{2}\sum_{n=1}^{R+1} n\mathbb{P}(S_n = 0) + (R+1-n)\mathbb{P}(S_{R+1} = n) \tag{11}$$

Split time process Conditionally to the trajectory S, the split time process is a series of independent exponential random variables:

$$\mathbb{P}(T_n = t | S_n = k) = ke^{-vkt}, \tag{12}$$

for $k > 0$. In the case $k = 0$, T is deterministic and equal to 0.
This means that the conditional expected value is $\mathbb{E}(T_n|S_n = k) = \frac{1}{vk}$.

The life span of a collective τ is given by the sum of all the splitting times T. Thus:

$$\begin{aligned}
\mathbb{E}(\tau) &= \sum_{n=1}^{R} \mathbb{E}(T_n) && (13)\\
&= \sum_{n=1}^{R}\sum_{k=0}^{n} \mathbb{E}(T_n|S_n = k)\mathbb{P}(S_n = k) && (14)\\
&= \frac{1}{v}\sum_{n=1}^{R}\sum_{k=1}^{n} \frac{1}{k}\mathbb{P}(S_n = k) && (15)\\
&= \frac{1}{v}\sum_{n=1}^{R}\frac{1}{n}\sum_{k=1}^{n} \binom{n}{\frac{n+k}{2}} p^{\frac{n+k}{2}-1} q^{\frac{n-k}{2}} && (16)
\end{aligned}$$

A.2 Special values

For $p = 0$, the first event is always a particle dispersal:

$$\mathbb{E}(\tau) = \frac{1}{v} \tag{17}$$

$$\rho = 1 \text{ (with probability 1)} \tag{18}$$

For $p = 1$, no propagule is produced and the collective life span is just the harmonic series:

$$\mathbb{E}(\tau) = \frac{1}{v}\sum_{k=1}^{R}\frac{1}{k}, \tag{19}$$

$$\rho = 0 \text{ (with probability 1).} \tag{20}$$

B Density-dependent p

Let $p : n \to p(n)$ be a function of n, the number of particles in the patch.

$$\mathbb{P}(S_n = i+1 | S_{n-1} = i) = p(i) \tag{21}$$

$$\mathbb{P}(S_n = i-1 | S_{n-1} = i) = 1 - p(i) \tag{22}$$

Let M be the transition matrix of the process S, such that $\mathbb{P}(S_n = i|S_0 = j) = M^n_{j,i}$. From Equation 11, we have:

$$\mathbb{E}(\rho) = \frac{1}{2} \sum_{n=1}^{R+1} n M_{1,0}^{n} + (R+1-n) M_{1,i}^{n}, \tag{23}$$

with

$$M = \begin{pmatrix} 1 & 0 & 0 & 0 & \dots & 0 \\ 1-p(1) & 0 & p(1) & 0 & \dots & 0 \\ 0 & 1-p(2) & 0 & p(2) & \dots & 0 \\ & & \dots & & & \\ 0 & 0 & 0 & 0 & \dots & 1 \end{pmatrix} \tag{24}$$

Supplementary Note 4 Conditions for Endogenisation

Consider two environmental values, called scaffolding e_s and non-scaffolding e_u. Now, $\tilde{\rho}$ and $\tilde{\tau}$ are functions of the underlying trait θ and the environment e. We assume that they are continuous.

Endogenisation relies on the existence of a hysteresis cycle as follows, when the four following conditions are fulfilled.

- **Condition 1 (non-scaffolded ESS)**. There exists an ancestral pre-ETI trait state θ_u and a post-ETI state θ_m, which are both evolutionarily stable in the non-scaffolding environment (the subscript stands for "unicell" and "multicell"). Without loss of generality, assume that $\theta_u < \theta_m$. It means that the function $\theta \mapsto \tilde{\rho}(\theta, e_u)$ has two local maxima in θ_u and θ_m. Since $\tilde{\rho}$ is continuous, there exists a local minimum θ_- such that $\theta_u < \theta_- < \theta_m$. Namely:

$$\begin{cases} \frac{\partial \tilde{\rho}(\theta_u, e_u)}{\partial \theta} = 0, & \frac{\partial^2 \tilde{\rho}(\theta_u, e_u)}{\partial \theta^2} < 0, \\ \frac{\partial \tilde{\rho}(\theta_m, e_u)}{\partial \theta} = 0, & \frac{\partial^2 \tilde{\rho}(\theta_m, e_u)}{\partial \theta^2} < 0, \\ \frac{\partial \tilde{\rho}(\theta_-, e_u)}{\partial \theta} = 0, & \frac{\partial^2 \tilde{\rho}(\theta_-, e_u)}{\partial \theta^2} > 0. \end{cases} \tag{25}$$

- **Condition 2 (scaffolded ESS)**. There exists an evolutionarily stable state θ_s in the scaffolding environment:

$$\frac{\partial \tilde{\rho}(\theta_s, e_s)}{\partial \theta} = 0, \; \frac{\partial^2 \tilde{\rho}(\theta_s, e_s)}{\partial \theta^2} < 0. \tag{26}$$

  For simplicity, we will consider that it is unique, but it would be enough to have its basin of attraction include both θ_u and θ_m.

- **Condition 3 (ecological stability)**. The population must be viable for the 3 ESS:

$$\begin{cases} \tilde{\rho}(\theta_u, e_u) > 1, \\ \tilde{\rho}(\theta_m, e_u) > 1, \\ \tilde{\rho}(\theta_s, e_s) > 1. \end{cases} \tag{27}$$

- **Condition 4 (ESS order)**. The scaffolded state must be in the basin of attraction of the post-ETI ESS:

$$\theta_u < \theta_- < \theta_s \tag{28}$$

Supplementary Figure 7 illustrates how the density-dependent duplication-dispersal ratio model fulfils these conditions.

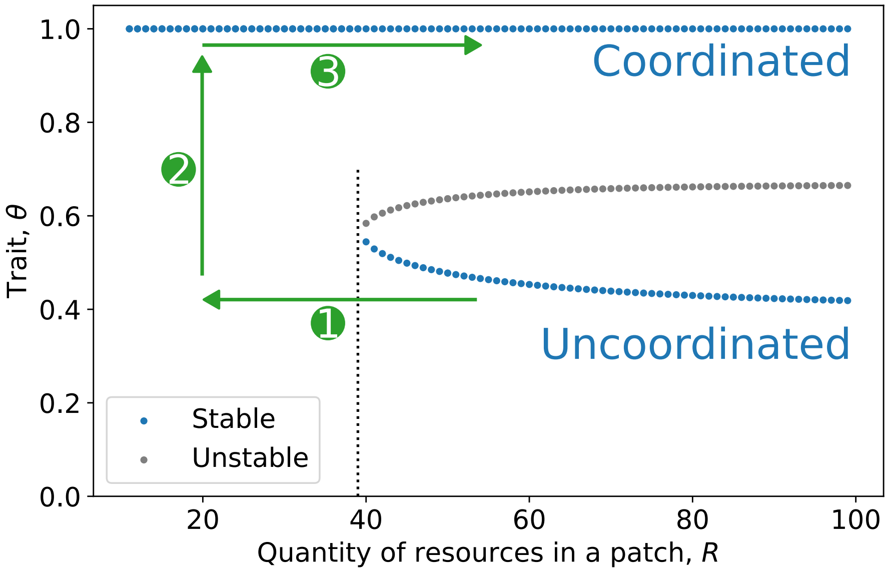

Supplementary Figure 7: **Singular evolutionary strategies for the density-dependent duplication-dispersal ratio as a function of R.** There are three branches of equilibria. Note the fold bifurcation at $R = 39$, the stable branch corresponding to uncoordinated particles (θ_u, bottom, stable), collides with the unstable branch (i.e., the bottom of the fitness valley, θ_-, in the middle), and they annihilate each other. The perfectly coordinated particle branch ($\theta_m = 1$, top) is always stable. If the system is on the bottom branch ($\theta_u < 0.5$, $R = 50$), a change from $e_u : R = 50$ to $e_s : R = 20$ (arrow 1, in green) causes the system to irremediably move to the top branch (arrow 2, $\theta_m = 1$, coordination between cells). If the environment is restored to $R = 50$ (arrow 3), the population does not revert to the ancestral phenotype. Such an irremediable change is called a hysteresis loop [4, 5].

Supplementary Note 5 Selection for Collective Size

Selection for collective size is a major theme in the study of evolutionary transitions [6]. However, the model presented in the main text assumes that there is no mechanism favouring larger collective size. This is due to simplifying assumptions, such as ignoring deaths within patches, that make the model amenable to numerical analysis. In this section, we relax several of these assumptions and explore the consequences for the size of collectives.

Since there is no advantage for a collective to contain more than two particles (i.e., one that stays to occupy the patch and the other that would disperse), the number of particles in each collective at the evolutionary equilibrium for the density-dependent dispersal probability model is quite small. On average, the number of particles is between one and two (See Figure **??**, and Supplementary Figure 9b). Indeed, the most efficient proto-organisms can get away with such a small soma size (because a bigger investment in soma does not translate into an increase in viability) and invest all remaining resources in fertility.

It is important to note, however, that making the probabilities change more smoothly between the value for a single cell in isolation and the value within a collective immediately increases the number of cells within a patch at evolutionary equilibrium. This can, for instance, be accomplished by taking the probability to duplicate to be $p(n,k) = (1 + \theta(n^k - 2))/n^k$ (with $k > 0$) in contrast to θ if n=1 and $1 - \theta$ otherwise.

Comparison of two trajectories demonstrates this. The first trajectory relies on the probabilities discussed in the main text (Supplementary Figure 8a), where birth and dispersal alternate perfectly (49 propagules on average, with an average mean number of 1.5 and an average maximum number of 2, Supplementary Figure 9a–b). The second trajectory instead uses smoothed probabilities (Supplementary Figure 8b), which allow for several births in succession. The average maximum number of particles within a collective is higher (at 3.8 particles, for 48 propagules, Supplementary Figure 9a–b) for the second trajectory.

Other small changes to the probability function can be implemented to obtain collectives of even larger size [1]. However, this often results in the loss of the hysteresis in the system: the local uncoordinated equilibrium disappears and highly coordinated collectives become the global attractor even in non-scaffolded conditions.

The interplay between collective size and the hysteresis cycle warrants an in-depth investigation, the likes of which is beyond the scope of the current manuscript. We conjecture that, for simple traits (e.g., one dimensional, density-dependent probability of dispersal without explicit consideration of internal cell states, delays, or transduction mechanisms), the existence of hysteresis requires small collective sizes. However, hysteresis cycles involving larger collective sizes could occur in more complex models.

References

1. Kiss, I. Z., Miller, J. C. & Simon, P. L. *Mathematics of Epidemics on Networks: From Exact to Approximate Models* ISBN: 978-3-319-50804-7 978-3-319-50806-1 (Springer International Publishing, Cham, 2017).
2. Geritz, S. A. H., Kisdi, E., Meszéna, G. & Metz, J. a. J. Evolutionarily singular strategies and the adaptive growth and branching of the evolutionary tree. *Evolutionary Ecology* **12,** 35–57 (1998).
3. Athreya, K. B. & Ney, P. E. *Branching Processes* ISBN: 978-3-642-65373-5 978-3-642-65371-1 (Springer Berlin Heidelberg, Berlin, Heidelberg, 1972).
4. Noori, H. R. in *Hysteresis Phenomena in Biology* (ed Noori, H. R.) 35–45 (Springer, Berlin, Heidelberg, 2014). ISBN: 978-3-642-38218-5.

[1] Inspired by the quorum-sensing mechanism, which is binary but not precise at the single cell level, we can change the probability so that there is a threshold between $p(n,t) = \theta$ for $\theta > k$ and $1 - \theta$ otherwise. This increases the size of the collectives at equilibrium. However, this destroys the hysteresis behaviour of the model.

5. Beisner, B., Haydon, D. & Cuddington, K. Alternative stable states in ecology. *Frontiers in Ecology and the Environment* **1,** 376–382 (2003).
6. Bonner, J. T. The origins of multicellularity. *Integrative Biology: Issues, News, and Reviews* **1,** 27–36 (1998).

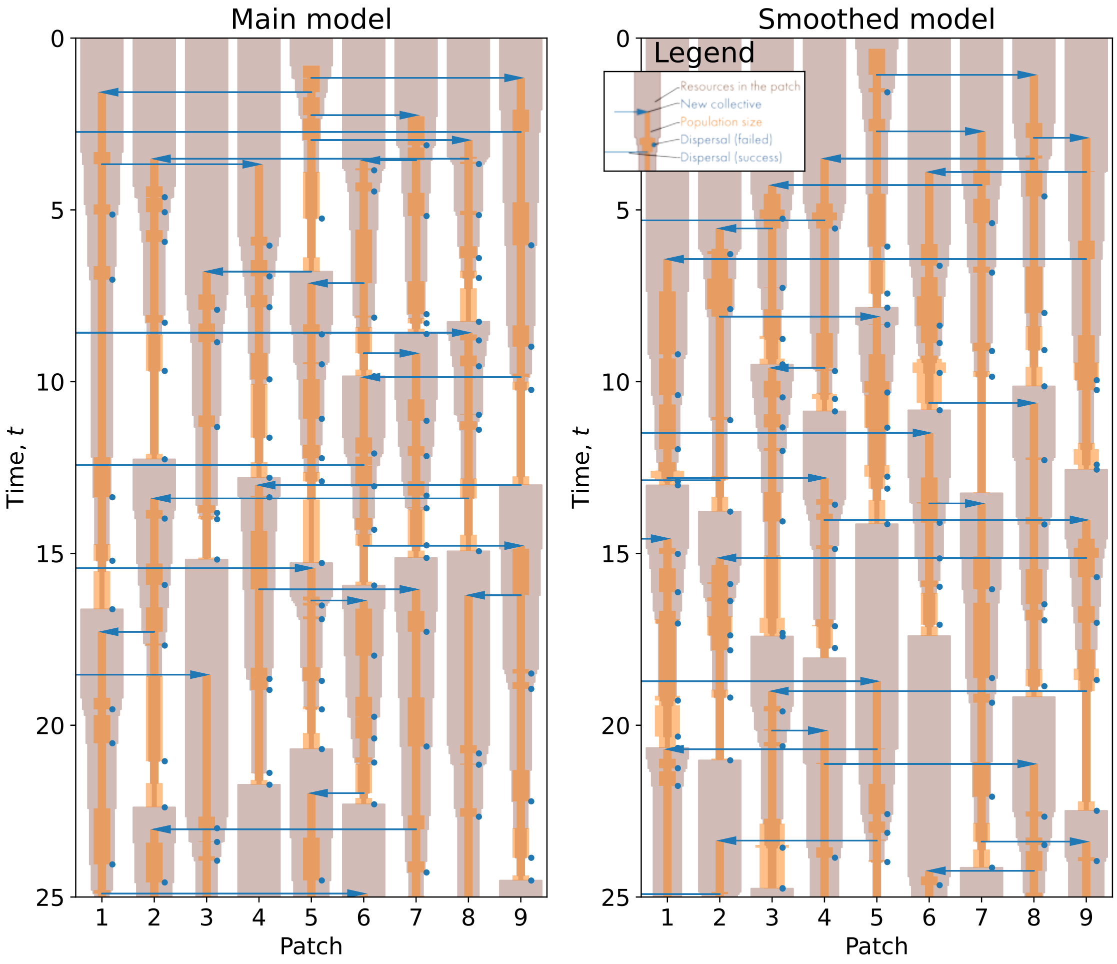

Supplementary Figure 8: **The smoothed-probability model displays higher collective sizes**. Each column corresponds to a collective. Time flows downwards. The brown area represents the available resources, the orange area represents the population of particles, blue lines and dots represent propagules (see legend). The density-dependent dispersal probability model presented in the main text for $\theta = 1$ is shown on the left. Note that collectives alternate between one and two particles. The same trajectories with smoothed probability (see main text) are depicted on the right. Note how higher collective sizes are reached.

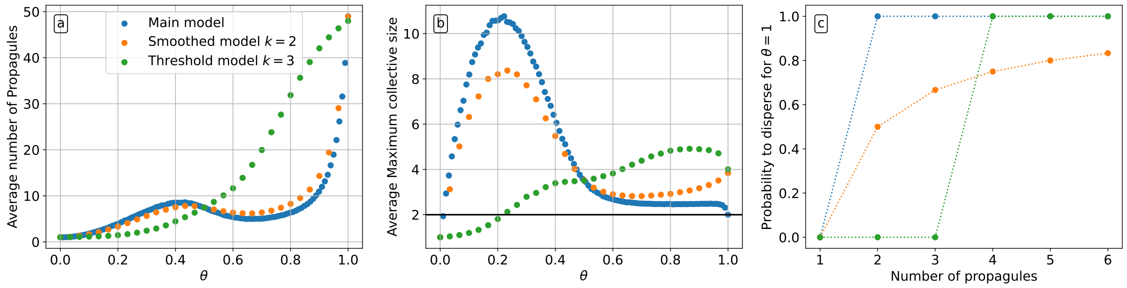

Supplementary Figure 9: **Size of collectives**. **a** Average (100, 000 simulations) number of propagules sent from a single collective for three models of probability of dispersal (see main text). **b** Average maximum collective size for the same simulations. **c** Probability for a particle to disperse given the number of particles on the patch for the three models.
