# Supplementary material for: Stability of ecologically scaffolded traits during evolutionary transitions in individuality
Source: Nat Commun. 2024 Aug 3;15:6566. doi: 10.1038/s41467-024-50625-1 (PMC11297203; doi:10.1038/s41467-024-50625-1)
Supplement: Supplementary file 3 — Description of Additional Supplementary Files [file 41467_2024_50625_MOESM3_ESM.pdf]

Description of Additional Supplementary Files

File Name: Supplementary Code 1
Description: The code and outputs used to generate all the figures and supplementary figures. Also hosted on Zenodo. https://doi.org/10.5281/zenodo.12582168
