# Supplementary material for: Stability of ecologically scaffolded traits during evolutionary transitions in individuality
Source: Nat Commun. 2024 Aug 3;15:6566. doi: 10.1038/s41467-024-50625-1 (PMC11297203; doi:10.1038/s41467-024-50625-1)
Supplement: Supplementary file 4 — Supplementary Code 1 [file 41467_2024_50625_MOESM4_ESM.zip › Description of Additional Supplementary Files.pdf]

Supplementary code for “Stability of Ecologically Scaffolded Traits During Evolutionary Transitions in Individuality”

This archive provides Supplementary code for: Doulcier, G. Takacs, P. Hammerschmidt, K. and Bourrat, P. Stability of Ecologically Scaffolded Traits During Evolutionary Transitions in Individuality. Nature Communications (2024).

It is organized in two folders:

- *scaffold/*: the simulation code in the format of a python package,
- *results/*: the results of the article.

In more details, the *results* folder contains:

- The figures used for the article in *results/fig*.
- The raw numerical data for each figure in *results/source_data*.
- All the code generating the figures as ipython notebooks. For convenience, all the notebooks are also exported as pdf in *result/notebook_exports*. This is where you should start if you want to review the code without installing the package yourself.
- The code for long simulations to be run using a job scheduler (SLURM) on a computer cluster. The output of the cluster jobs is also provided in `https://doi.org/10.5281/zenodo.12582168`.
- The exact version of all packages used to generate the results in *python-env.txt*.
