# Supplementary material for: Stability of ecologically scaffolded traits during evolutionary transitions in individuality
Source: Nat Commun. 2024 Aug 3;15:6566. doi: 10.1038/s41467-024-50625-1 (PMC11297203; doi:10.1038/s41467-024-50625-1)

Notebook 01_evoCstR.ipynb

Guilhem Doulcier

June 28, 2024

This notebook parse the result of the script `01_evoRcst.py` and create the figure S3.

[1]:
```
import glob
import os

import matplotlib.pyplot as plt
import scaffold.meanfield.analytical
import scaffold.network.reader
import scaffold.network.recorder
from scaffold import labels
from scipy.optimize import fmin

!mkdir -p source_data
!mkdir -p fig/supfig
```

[2]:
```
path = os.path.join(os.getcwd(),"output","evoRcst3","*.json")
print(len([file for file in glob.glob(path)]), "simulation outputs to parse")
# Import and read the raw dataset
df = scaffold.network.reader.extract_folder(path, cache=True)
df.loc[df.ecology_name =="threshold","mean_trait"] = 1 - df[df.ecology_name␣
↪=="threshold"].mean_trait
```

```
239 simulation outputs to parse
```

[3]:
```
# Compute the maximum value of rho.
popt = {}
for R in df.R.unique():
    popt[R] = fmin(lambda p: -scaffold.meanfield.analytical.rho(p,R), 0.5,␣
↪disp=False)
```

[4]:
```
e_space = sorted(df.ecology_name.unique())
r_space = sorted(df.R.unique())
plt.rc('font', size=15)
fig,ax = plt.subplots(2, 2, figsize=(15,7))

for i,e in enumerate(e_space):
    for j,R in enumerate(r_space):
        datum = df[df.ecology_name==e]
        datum = datum[datum.R==R]
```

```
        if 'constant'  in e:
            t = "Intrinsic duplication-dispersal"
            ax[j,i].hlines(popt[R],0,df.e.max(),color='k',
                           ls='--', label=f'R={R} - {e}', zorder=99)
        else:
            t = "Density dependent duplication-dispersal"

        ax[j,i].text(0.99,0.99,f'{t}, R = {R}', horizontalalignment='right',
                     verticalalignment='top',
                     transform=ax[j,i].transAxes)

        ax[j,i].text(0.04,0.96,'abcd'[j+i*2], horizontalalignment='right',
             verticalalignment='top', fontweight='bold',
             bbox=dict(facecolor='none', edgecolor='black',␣
 ↪boxstyle='round,pad=0.2'),
             transform=ax[j,i].transAxes)
        datum.to_csv('source_data/s3{}_constantR_trajectory.csv'.
 ↪format('abcd'[j+i*2]))
        for k, d in datum.groupby('simulation_id'):
            ax[j,i].plot(d.e, d['mean_trait']
                         , color='C0' if R==100 else 'C1')
        ax[i,j].set(ylim=[-0.01,1.10], )
plt.rc('font', size=15)
ax[0,0].set(ylabel=labels['trait'], )
ax[1,1].set(xlabel=labels['time'])
ax[1,0].set(ylabel=labels['trait'], xlabel=labels['time'])
plt.tight_layout()

fig.savefig('fig/supfig/s3_constantR_trajectory.svg')
fig.savefig('fig/supfig/s3_constantR_trajectory.pdf')
```

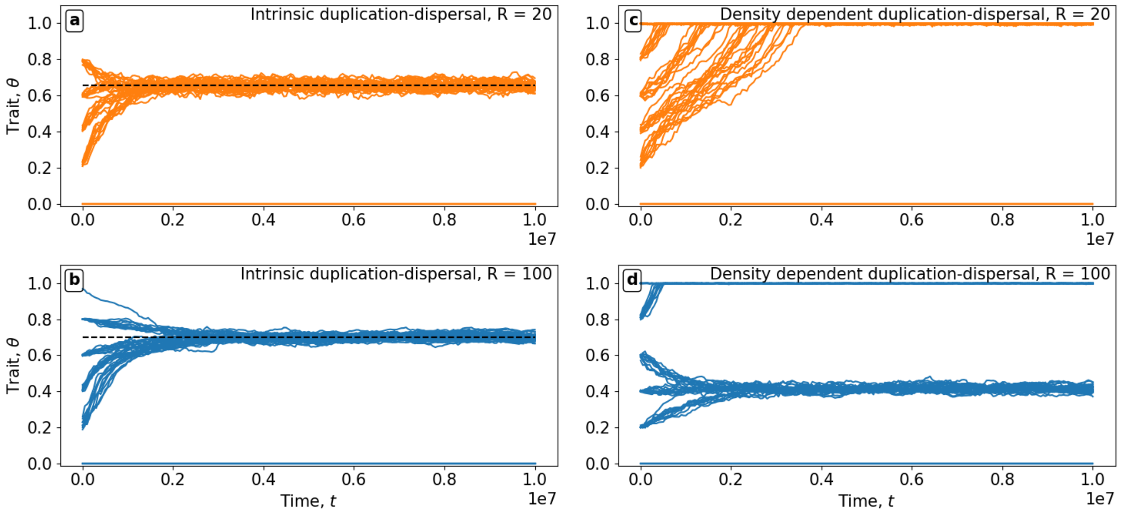

Supplement: Supplementary file 4 — Supplementary Code 1 [file 41467_2024_50625_MOESM4_ESM.zip › code/results/notebook_exports/01_evoCstR.pdf]
