# Supplementary material for: Stability of ecologically scaffolded traits during evolutionary transitions in individuality
Source: Nat Commun. 2024 Aug 3;15:6566. doi: 10.1038/s41467-024-50625-1 (PMC11297203; doi:10.1038/s41467-024-50625-1)

Notebook 02_scaffold_clamp.ipynb

Guilhem Doulcier

June 28, 2024

This notebook parse the result of the `02_scaffold_clamp.py` script and produce Fig 4e and 4d

[1]:
```
import glob
import os

import matplotlib.pyplot as plt
import numpy as np
import pandas as pd
import scaffold.network.reader
import scaffold.network.recorder
from scaffold import labels

!mkdir -p source_data
!mkdir -p fig
```

[2]:
```
path = os.path.join(os.getcwd(),"output","scaffold_clamp3","*.json")
print(len([file for file in glob.glob(path)]), "simulation outputs to parse")
df = scaffold.network.reader.extract_folder(path, cache=True)
df.loc[df.ecology_name =="threshold","mean_trait"] = 1 - df[df.ecology_name␣
↪=="threshold"].mean_trait
```

```
148 simulation outputs to parse
```

[3]:
```
raw_points = []
for i, d in df.groupby('simulation_id'):
    d['scaffolded'] = d.R_field==d.Rscaffolded
    t_start =  d.loc[d.scaffolded].time.min()
    t_end =  d.loc[d.scaffolded].time.max()
    plt.scatter(t_end-t_start, d.mean_trait.iloc[-1],color='C0', marker='.')
    raw_points.append({'Duration':t_end-t_start, 'Final mean trait value': d.
↪mean_trait.iloc[-1]})


plt.gca().set(xlabel="Duration of the scaffolding, T",
              ylim=(0,1.05),
            ylabel="Final  "+labels['mean_trait'])
pd.DataFrame(raw_points).to_csv('source_data/4e_scaffold_clamp_final_trait_by_T.
↪csv')
```

```
plt.savefig("fig/4e_scaffold_clamp_final_trait_by_T.svg")
plt.savefig("fig/4e_escaffold_clamp_final_trait_by_T.svg")
```

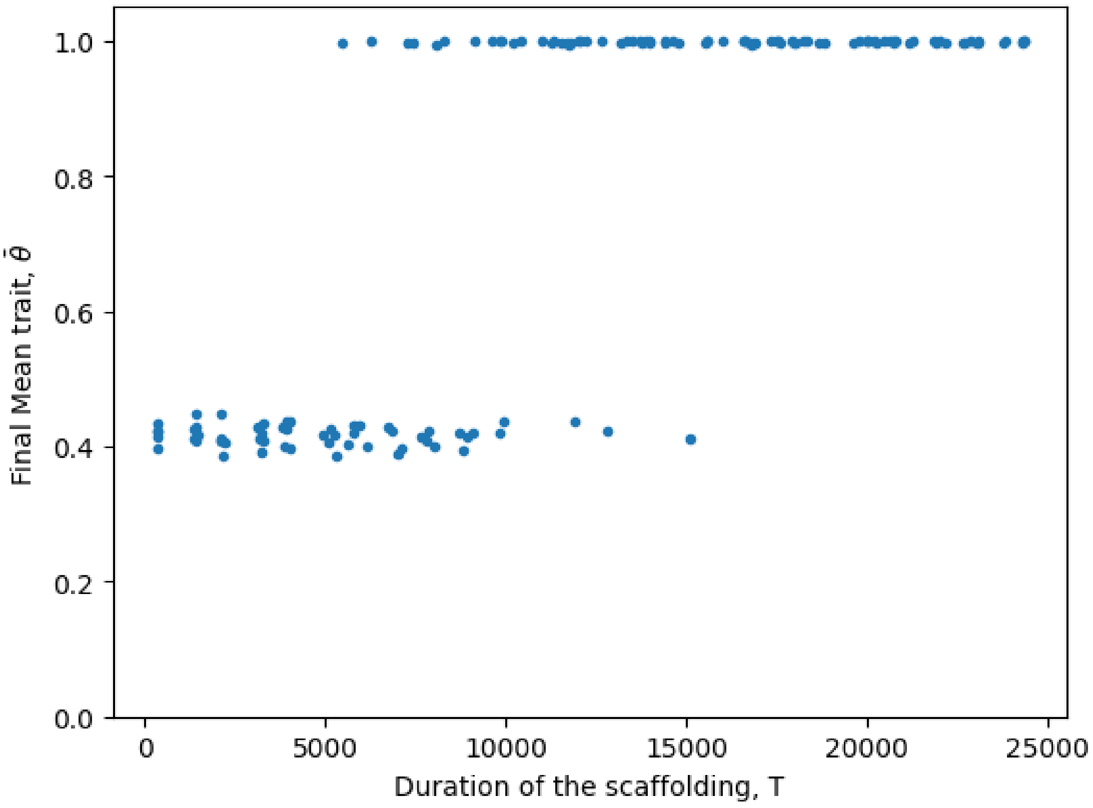

[4]:
```
fig, ax = plt.subplots(25, 1, figsize=(2.7,25))
trait_space = [0,1]
trait_range = [np.min(trait_space), np.max(trait_space)]
df['scaffolded'] = df.R_field==df.Rscaffolded
for u,(k,dd) in enumerate(df.groupby('Tendclamp')):
    for k,d in dd.groupby('simulation_id'):
        me = d[d.scaffolded].e.min()
        ax[u].plot(d.time[d.e<=me], d.mean_trait[d.e<=me], color='C0')
        ax[u].plot(d.time[d.R_field==d.Rscaffolded], d.mean_trait[d.R_field==d.
↪Rscaffolded], color='C1')
        ax[u].plot(d.time[d.e>=d.Tendclamp], d.mean_trait[d.e>=d.Tendclamp],␣
↪color='C0')
    ax[u].plot([],[], color='C0', label="Unscaffolded: R=20")
    ax[u].set(ylim=(0.3,1.05), xlim=(0,50000))
    if u<3:
        ax[u].set(xticklabels=[], ylabel=labels['mean_trait'])
    ax[u].plot([],[], color='C1', label="Scaffolded: R=100")
```

```
ax[-1].legend()
ax[-1].set(xlabel=labels['time'], ylabel=labels['mean_trait'],)
plt.tight_layout()
df[['scaffolded','e','simulation_id']].to_csv('source_data/
    ↪4d_scaffold_trajectory_separated.csv')
fig.savefig('fig/4d_scaffold_trajectory_separated.svg', bbox_inches='tight')
fig.savefig('fig/4d_scaffold_trajectory_separated.png', bbox_inches='tight')
```

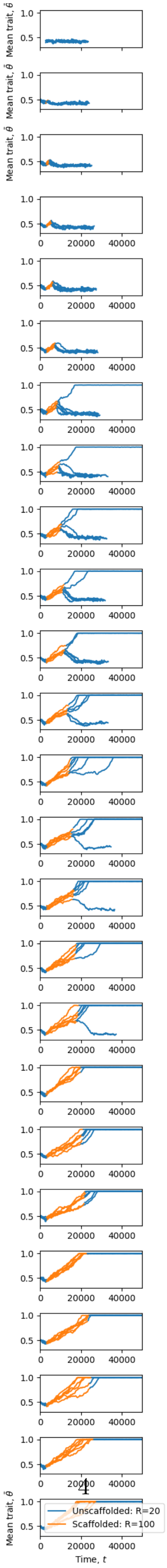

Mean trait, θ̄
0.5
1.0
0
20000
40000
Time, t
Unscaffolded: R=20
Scaffolded: R=100

[5]:
```python
fig, ax = plt.subplots(1, 1, figsize=(12,5))
trait_space = [0,1]
trait_range = [np.min(trait_space), np.max(trait_space)]
for k,d in df.groupby('simulation_id'):
    if True:
        d['scaffolded'] = d.R_field==d.Rscaffolded
        me = d[d.scaffolded].e.min()

        ax.plot(d.time[d.e<=me], d.mean_trait[d.e<=me], color='C0')
        ax.plot(d.time[d.R_field==d.Rscaffolded], d.mean_trait[d.R_field==d.
 ↪Rscaffolded], color='C1')
        ax.plot(d.time[d.e>=d.Tendclamp], d.mean_trait[d.e>=d.Tendclamp],␣
 ↪color='C0')

ax.plot([],[], color='C0', label="Unscaffolded: R=20")
ax.plot([],[], color='C1', label="Scaffolded: R=100")
ax.legend()
ax.set(xlabel=labels['time'], ylabel=labels['mean_trait'], xlim=(0,40000))
```

[5]:
```
[Text(0.5, 0, 'Time, $t$'),
 Text(0, 0.5, 'Mean trait, $\\bar \\theta$'),
 (0.0, 40000.0)]
```

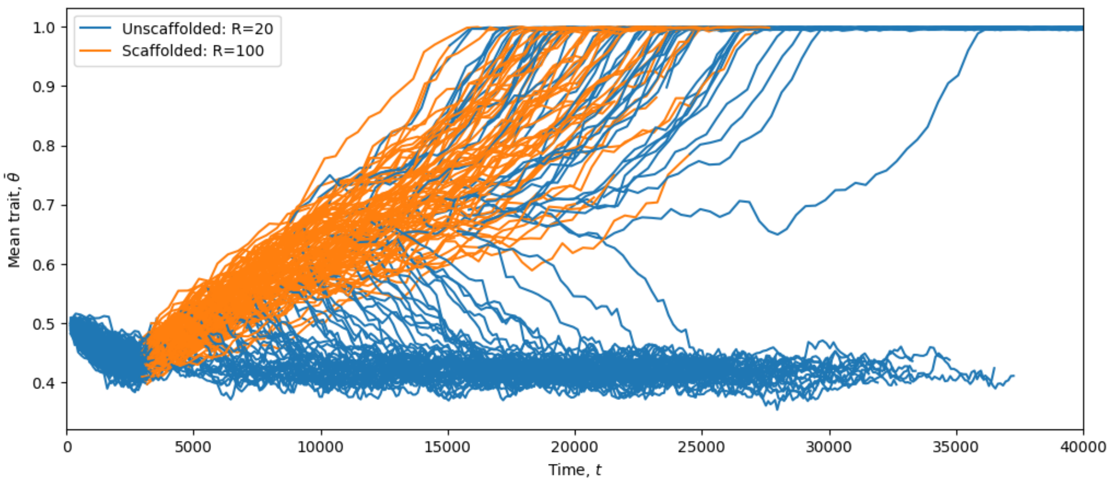

Supplement: Supplementary file 4 — Supplementary Code 1 [file 41467_2024_50625_MOESM4_ESM.zip › code/results/notebook_exports/02_scaffold_clamp.pdf]
