# Supplementary material for: Stability of ecologically scaffolded traits during evolutionary transitions in individuality
Source: Nat Commun. 2024 Aug 3;15:6566. doi: 10.1038/s41467-024-50625-1 (PMC11297203; doi:10.1038/s41467-024-50625-1)
Supplement: Supplementary file 4 — Supplementary Code 1 [file 41467_2024_50625_MOESM4_ESM.zip › code/results/notebook_exports/03_gradient.pdf]

Notebook 03_gradient.ipynb

Guilhem Doulcier

June 28, 2024

This notebook parse the result of the `03_gradient.py` script and produces fig 5.

[1]:
```
import glob
import os

import matplotlib.pyplot as plt
import numpy as np
import pandas as pd
import scaffold.meanfield.analytical
import scaffold.network.reader
import scaffold.network.recorder
from mpl_toolkits.axes_grid1 import make_axes_locatable
from scaffold import labels

plt.rc('font', size=15)
fig_path = 'fig'
!mkdir -p {fig_path}
!mkdir -p source_data
```

[2]:
```
def extract_trajectory(trajectory_list):
    N = len(trajectory_list)
    for t, entry in enumerate(trajectory_list):
        field = scaffold.network.recorder.npzstr_to_field(entry['field'])
        #print(field.event, field.time)
        if not t:
            phenotypes = field.phenotypes
            density = np.zeros((len(phenotypes),N), dtype=int)
            index_R = {R:i for i,R in enumerate(sorted(pd.Series(field.renewal).
 ↪unique()))}
            density_R = np.zeros((len(index_R),N), dtype=int)
            trait_R = np.zeros((len(index_R),N))
            pop = np.zeros(N, dtype=int)
            mean = np.zeros(N)
            time = np.zeros(N)
            events = np.zeros(N)
        time[t] = field.time.copy()
        events[t] = field.event.copy()
        u = len(field.phenotypes)
```

```
        for i,p in enumerate(field.phenotypes):
            i = u-i-1
            density[i,t] = field.ind[p].sum()
            pop[t] += density[i,t]
            mean[t] += density[i,t]*p
            for j,R in enumerate(field.renewal):
                density_R[index_R[R],t] += field.ind[p][j]
                trait_R[index_R[R],t] += field.ind[p][j]*(1-p)
    mean[pop!=0] /= pop[pop!=0]
    trait_R[density_R!=0] /= density_R[density_R!=0]
    mean[pop==0] = np.nan
    trait_R[density_R==0] = np.nan
    return {"phenotype":1-phenotypes,
            "density":density,
            "pop":pop,
            "mean":1-mean,
            "time":time,
            "event":events,
            "density_R":density_R,
            "trait_R":trait_R}
```

[3]:
```
path = os.path.join(os.getcwd(),"output","Rgradient7","output_*.json")
meta = []
rawdata = []

for file in glob.glob(path):
    name = os.path.basename(file)
    raw,comment = scaffold.network.recorder.load_trajectory(file)
    comment['filename'] = name
    meta.append(comment)
    rawdata.append(raw)

data = [extract_trajectory(raw) for raw in rawdata]
metadata = pd.DataFrame(meta)
metadata
```

[3]:

	mut	mix	n_traits	initial_trait	Rmin	Rmax	Rstep	rows	Tend	\
0	0.01	False	101	0.5	2	100	1	10	200000000	

	ecology_name	save_period	simulation_id	filename
0	threshold	50000	2	output_simu_2.json

[4]:
```
def display_experiment(data):
    fig,ax = plt.subplots(3,1,figsize=(10,3*5))

    viridis = plt.get_cmap('viridis_r')
    viridis.set_under('white')
```

```
    plasma = plt.get_cmap('plasma')
    plasma.set_under('white')

    def make_colorbar_with_padding(ax):
        """
        Create colorbar axis that fits the size of a plot
        detailed here: http://chris35wills.github.io/matplotlib_axis/
        """
        divider = make_axes_locatable(ax)
        cax = divider.append_axes("right", size="1%", pad=0.1)
        return(cax)

    cax = [make_colorbar_with_padding(a) for a in ax]
    ax[0].set(ylabel=labels['trait'], xlabel=labels['time'])
    ax[0].set(xticklabels=[], ylim=(0.3,1))
    m = ax[0].imshow(data['density'], cmap=viridis,aspect='auto',␣
 ↪origin='lower',
                 extent=(0,data['density'].shape[0],0,1), vmin=1,␣
 ↪interpolation='none')
    plt.colorbar(m, cax[0], ax[0], label='Number of Individuals')
    ax[1].set(ylabel=labels['R'], xticklabels=[], xlabel=labels['time'])
    m = ax[1].imshow(data['density_R'], cmap=viridis,aspect='auto',␣
 ↪origin='lower',
                 interpolation='none', vmin=1)
    plt.colorbar(m, cax[1], ax[1], label='Mean number\n of individuals in a␣
 ↪patch')

    ax[2].set(ylabel=labels['R'],xticklabels=[], xlabel=labels['time'])
    m = ax[2].imshow(data['trait_R'], cmap=plasma, aspect='auto',␣
 ↪origin='lower', interpolation='none')
    plt.colorbar(m, cax[2], ax[2], label=r'Mean trait $\theta$')
    return fig, ax
```

[5]:
```
for meta, datum in zip(metadata.iterrows(), data):
    print(meta[1].filename)
    fig, ax = display_experiment(datum)
plt.tight_layout()

fig.savefig(os.path.join(fig_path, '5_gradient.png'), bbox_inches='tight',␣
 ↪dpi=300)
fig.savefig(os.path.join(fig_path, '5_gradient.svg'), bbox_inches='tight',␣
 ↪dpi=300)
pd.json_normalize(datum).to_csv('source_data/5_gradient.csv')
```

```
output_simu_2.json
```

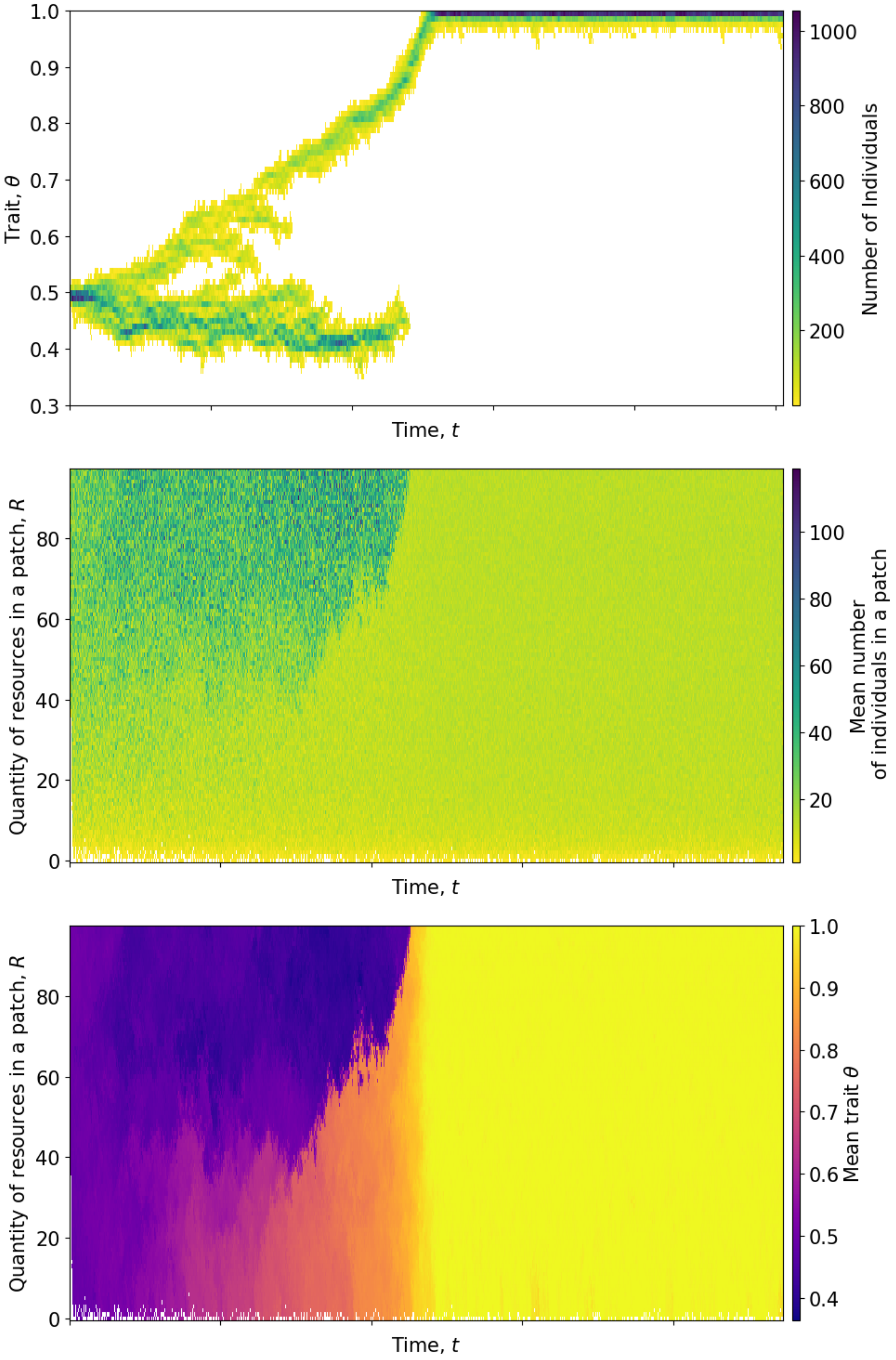
1.0
0.9
0.8
0.7
0.6
0.5
0.4
0.3
Trait, θ
Time, t
1000
800
600
400
200
Number of Individuals
80
60
40
20
0
Quantity of resources in a patch, R
Time, t
100
80
60
40
20
Mean number
of individuals in a patch
80
60
40
20
0
Quantity of resources in a patch, R
Time, t
1.0
0.9
0.8
0.7
0.6
0.5
0.4
Mean trait θ

[ ]:

```

```
