# Supplementary material for: Stability of ecologically scaffolded traits during evolutionary transitions in individuality
Source: Nat Commun. 2024 Aug 3;15:6566. doi: 10.1038/s41467-024-50625-1 (PMC11297203; doi:10.1038/s41467-024-50625-1)
Supplement: Supplementary file 4 — Supplementary Code 1 [file 41467_2024_50625_MOESM4_ESM.zip › code/results/notebook_exports/04_ODE.pdf]

Notebook 04_ODE.ipynb

Guilhem Doulcier

June 28, 2024

1 ODE Model

This notebook plot a few analytical and numerical resutls about the ODE system It prodeuces figure S2, S4, S6, 3b

[1]:
```
import os

import matplotlib.pyplot as plt
import numpy as np
import pandas as pd
import scaffold.meanfield.analytical as analytical
from scaffold import labels
from scipy.optimize import fmin

plt.rc('font', size=15)
fig_path = 'fig/supfig'
!mkdir -p {fig_path}
!mkdir -p source_data
```

1.1 Niche Occupancy from the ODE model

Compute the fraction of occupied niche as a function of the lifetime propagule production ρ.

[2]:
```
pspan = np.linspace(0,10,500)
occ = [analytical.occupancy_rho(p, D=1, d=1) for p in pspan]

fig, ax = plt.subplots(1,1, figsize=(8,4))
plt.hlines(0,1,pspan.max(), ls='--')
ax.plot(pspan, occ)
ax.set(xlabel=labels['rho'], xticks=np.arange(11),
       ylabel=labels['C*'], yticks=[0,.2,.4,.6,.8,1],
       yticklabels=["0%", "20%", "40%", "60%", "80%", "100%"])
fig.savefig(os.path.join(fig_path, "s4_occupancy_theo.svg"),␣
 ↪bbox_inches='tight')
fig.savefig(os.path.join(fig_path, "s4_occupancy_theo.pdf"),␣
 ↪bbox_inches='tight')
pd.DataFrame({'rho':pspan, "occupancy":occ}).to_csv("source_data/
 ↪s4_occupancy_theo.csv")
```

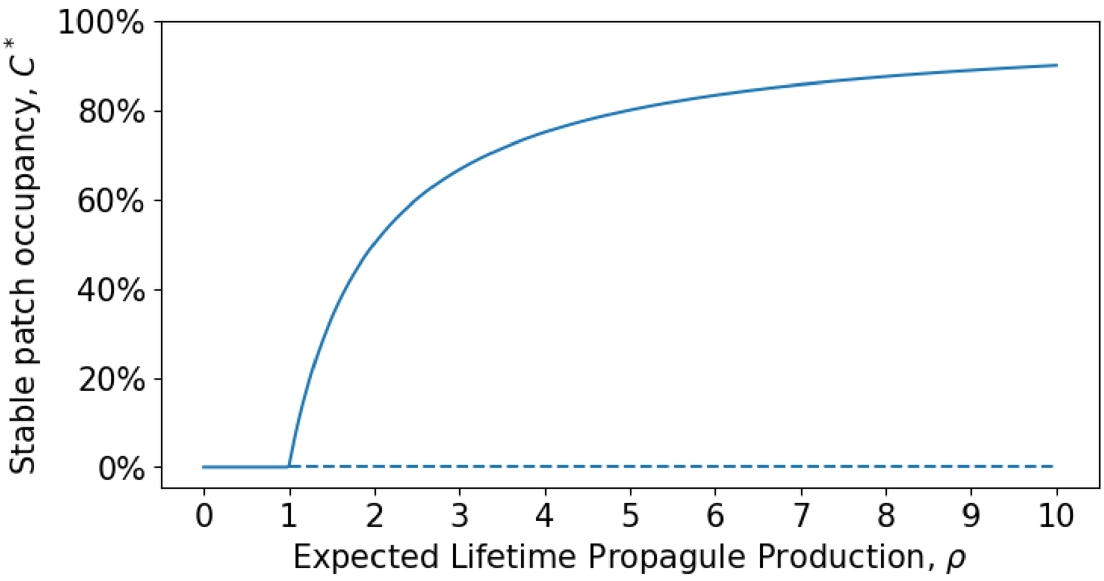

1.2 ESS for constant p

[3]:
```
Rspan = np.arange(2,100)
pstar = [fmin(lambda p: -analytical.rho(p,R), 0.5, disp=0) for R in Rspan]
```

[4]:
```
fig, ax = plt.subplots(1,1, figsize=(8,5))
ax.set(xlabel=labels['R'],
       ylabel=labels['theta'],
      title=r"Evolutionary Singular Strategy for $p(\theta)=\theta$")
ax.plot(Rspan, pstar)
fig.savefig(os.path.join(fig_path, "s6_ESS.svg"))
fig.savefig(os.path.join(fig_path, "s6_ESS.pdf"))
pd.DataFrame({'R':Rspan, "ess_trait_value":pstar}).to_csv("source_data/s6_ESS.
  ↪csv")
```

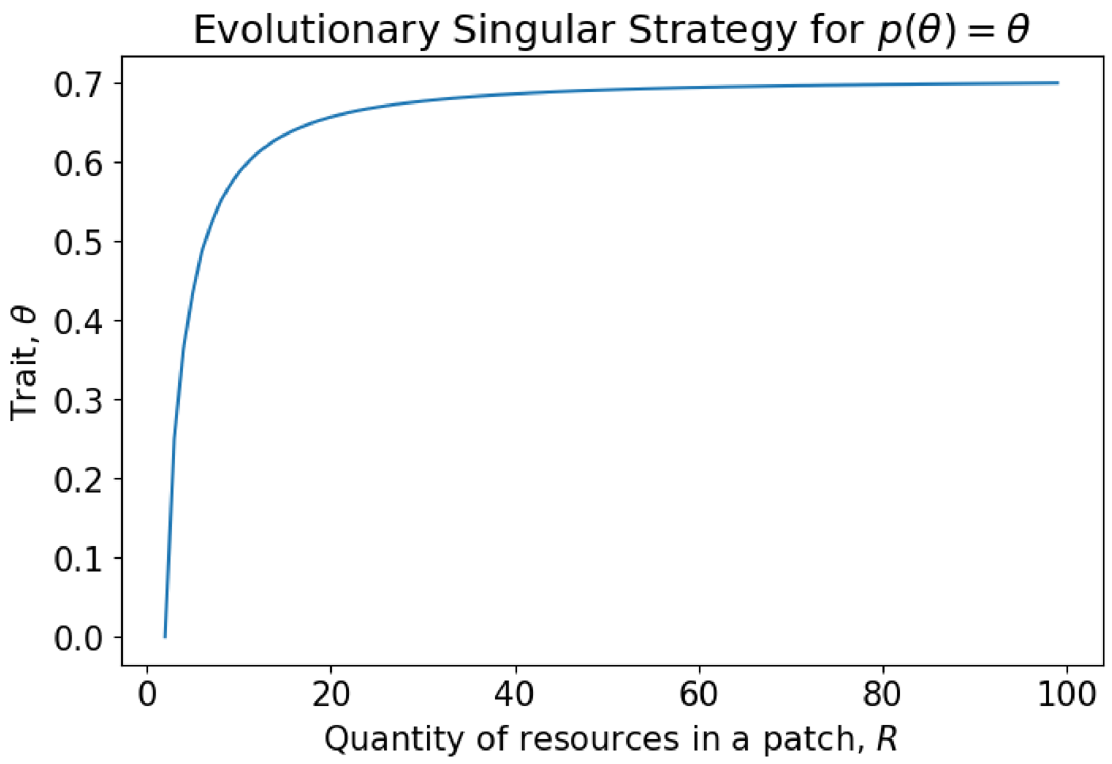

2 rho = f(p)

[5]:
```
fig, ax = plt.subplots(1,1, figsize=(8,5))
Rspan2 = [10,20,30,40,50]
pspan = np.linspace(0,1,100)

raw = pd.DataFrame({'trait':pspan})
for R in Rspan2:
    rho = [analytical.rho(p,R) for p in pspan]
    raw[f'rho_{R}'] = rho
    plt.plot(pspan,rho, label=f'R={R}')
ax.plot(pstar, [analytical.rho(p,R) for p,R in zip(pstar,Rspan)],color='k',␣
 ↪ls=':')
ax.legend()
ax.set(xlabel=labels['theta'],
       ylabel=labels['rho'],
      ylim=[-0, 12])
fig.savefig(os.path.join('fig', "3b_rho_pconstant.svg"))
fig.savefig(os.path.join('fig', "3b_rho_pconstant.pdf"))
raw.to_csv("source_data/3b_rho_pconstant.csv")
```

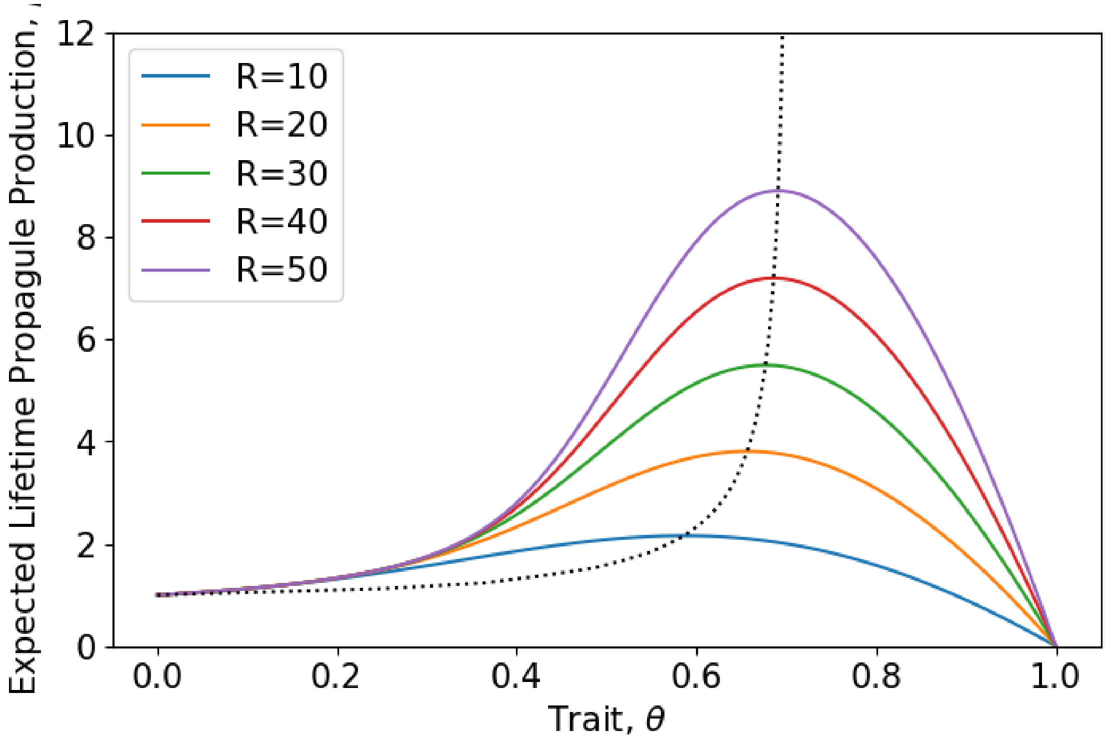

2.1 Rho-tau tradeoff

- Plots of rho and tau for different values of constant p.
- Check that the analytical derivative in scaffold.meanfield.analytical is correct.

[6]:
```
def stochastic(p,R):
    x = 1 # cells
    z = 0 # propagules
    t = 0 # time
    for _ in range(R):
        t += np.random.exponential(1/x)
        if np.random.random()<p:
            x += 1
        else:
            x -= 1
            z += 1
        if not x:
            break
    return t, x, z


import pandas as pd
```

```
def all_sim(R, repl, cache=True):
    filename = f"output/single_patch/single_patch_{R}_{repl}.csv"
    if cache and os.path.exists(filename):
        return pd.read_csv(filename)
    data_points = []
    for i, p in enumerate(np.linspace(0,1,30)):
        for _ in range(int(repl)):
            t, x, z = stochastic(p, R)
            data_points.append({"R":R, "p":p, "rho":z, "tau": t, "x":x})
    data = pd.DataFrame(data_points)
    data.to_csv(filename)
    return data
```

[7]:

```
from scaffold.meanfield.analytical import rho, tau

repl = int(1.5e5)
fig, ax = plt.subplots(2,2, figsize=(14,14))
raw =  pd.DataFrame({})
for i,R in enumerate([5, 50]):
    data = all_sim(R,repl)
    data_mean = data.groupby('p').mean().reset_index()
    data_std = data.groupby('p').std().reset_index()

    r = [rho(p, R) for p in pspan]
    t = [tau(p, R) for p in pspan]

    raw[f"rho_{R}_trait"] = data_mean.p
    raw[f"rho_{R}_mean"] = data_mean.rho
    raw[f"tau_{R}_mean"] = data_mean.tau
    raw[f"rho_{R}_std"] = data_std.rho
    raw[f"tau_{R}_std"] = data_std.tau

    ax[i,0].plot(pspan, r, color='C0', label=labels['rho'])
    ax[i,0].plot(pspan, t, color='C1', label=labels['tau'])
    std = False
    if std:
        ax[i,0].errorbar(data_mean.p,data_mean.rho,yerr=data_std.rho,␣
↪color='C0', marker='.', ls='')
        ax[i,0].errorbar(data_mean.p,data_mean.tau,yerr=data_std.tau,␣
↪color='C1', marker='.', ls='')
        ax[i,1].errorbar(data_mean.rho,data_mean.tau, xerr=data_std.rho,␣
↪yerr=data_std.tau, color='C2', ls='', marker='.')
    else:
        ax[i,0].scatter(data_mean.p,data_mean.rho, color='C0', marker='o')
        ax[i,0].scatter(data_mean.p,data_mean.tau, color='C1', marker='o')
        ax[i,1].scatter(data_mean.rho,data_mean.tau, color='C2', marker='o')
```

```
    ax[i,1].plot(r, t, color='C2')
    ax[i,0].legend()
    ax[i,1].set(xlabel=labels['rho'], ylabel=labels['tau'])

    ax[i,1].text(0.01 if i==0 else 0.4, 0.01,f'Points are average\nfrom {repl}␣
↪simulations', horizontalalignment='left',
             verticalalignment='bottom',
             transform=ax[i,1].transAxes)

    ax[i,1].text(0.01, 0.90,f'R={R}', horizontalalignment='left',
         verticalalignment='bottom',
         transform=ax[i,1].transAxes)
    ax[i,0].text(0.01, 0.90,f'R={R}', horizontalalignment='left',
     verticalalignment='bottom',
     transform=ax[i,1].transAxes)

    ax[i,0].set(xlabel=labels['theta'])
raw.to_csv('source_data/s2_tradeoff.csv')
plt.savefig('fig/supfig/s2_tradeoff.svg')
plt.savefig('fig/supfig/s2_tradeoff.pdf')
```

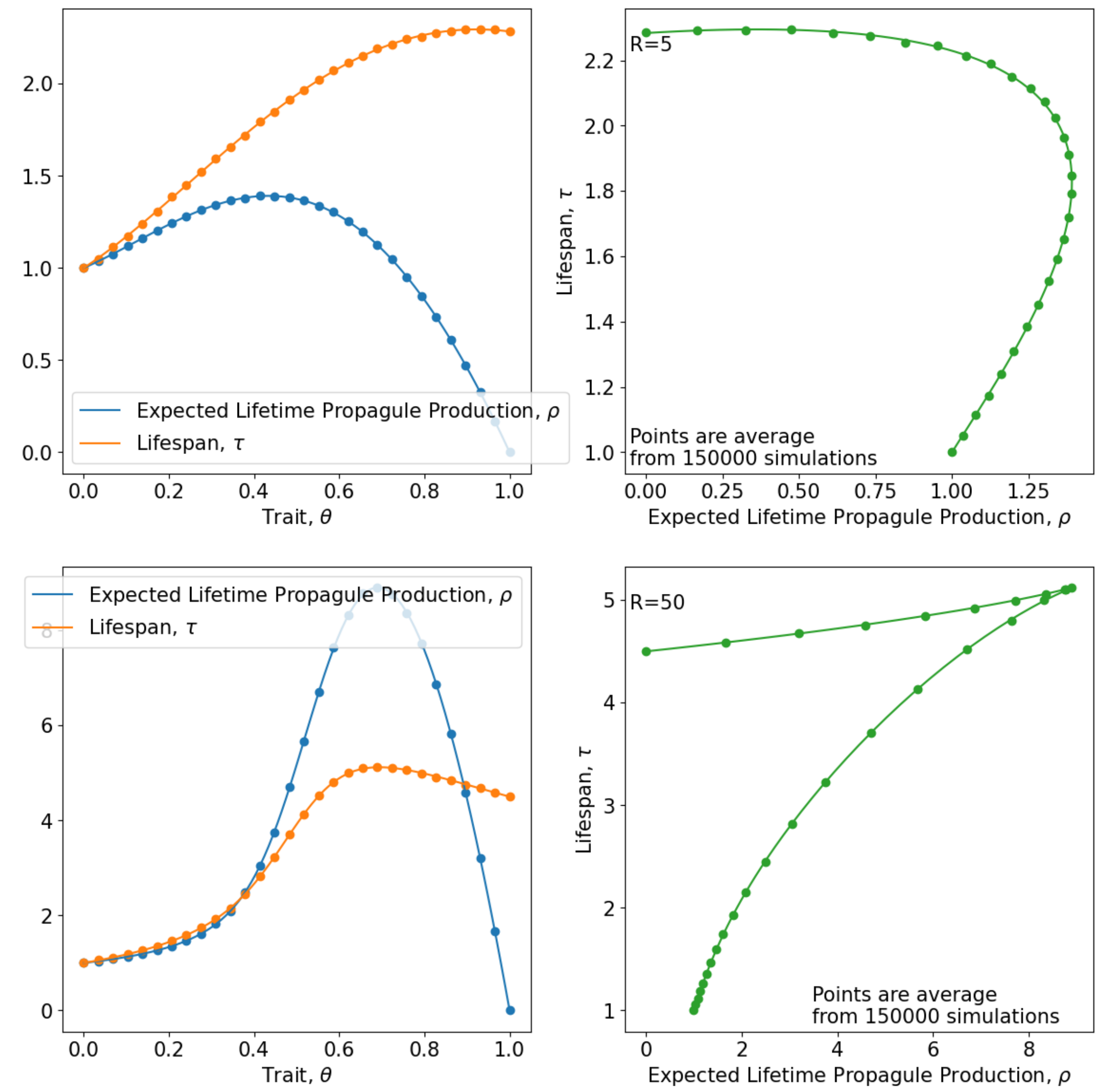

3 Extra figure: Derivatives

[8]:
```
from scaffold.meanfield.analytical import diff_rho, diff_tau, rho, tau
from scipy.misc import derivative

for N in [5, 50]:
    fig, ax = plt.subplots(1,2, figsize=(10,5))
    drho = lambda p : derivative(lambda x: rho(p=x,R=N), p, dx=1e-10)
    dtau = lambda p : derivative(lambda x: tau(p=x,R=N), p, dx=1e-10)

    pspan = np.linspace(0,1)[1:-1]
    tax0 = ax[0].twinx()
```

```
    tax1 = ax[1].twinx()

    tax0.plot(pspan, [rho(p, N) for p in pspan], color='C1', label='rho')
    tax1.plot(pspan, [tau(p, N) for p in pspan], color='C1', label='tau')

    ax[0].scatter(pspan, [drho(p) for p in pspan], label="Numerical derivation")
    ax[1].scatter(pspan, [dtau(p) for p in pspan], label="Numerical derivation")

    ax[0].plot(pspan, [diff_rho(p,N) for p in pspan], label="analytic␣
↪derivation")
    ax[1].plot(pspan, [diff_tau(p,N) for p in pspan], label="analytic␣
↪derivation")
    ax[0].set(title=rf"$d\rho/dp, N={N}$", xlabel='p')
    ax[1].set(title=rf"$d\tau/dp, N={N}$", xlabel='p')
    ax[0].legend()
    for a in ax:
        a.hlines(0, 0, 1, color='k')
    plt.tight_layout()
```

```
/tmp/ipykernel_81560/3731348846.py:6: DeprecationWarning: scipy.misc.derivative
is deprecated in SciPy v1.10.0; and will be completely removed in SciPy v1.12.0.
You may consider using findiff: https://github.com/maroba/findiff or
numdifftools: https://github.com/pbrod/numdifftools
  drho = lambda p : derivative(lambda x: rho(p=x,R=N), p, dx=1e-10)
/tmp/ipykernel_81560/3731348846.py:7: DeprecationWarning: scipy.misc.derivative
is deprecated in SciPy v1.10.0; and will be completely removed in SciPy v1.12.0.
You may consider using findiff: https://github.com/maroba/findiff or
numdifftools: https://github.com/pbrod/numdifftools
  dtau = lambda p : derivative(lambda x: tau(p=x,R=N), p, dx=1e-10)
```

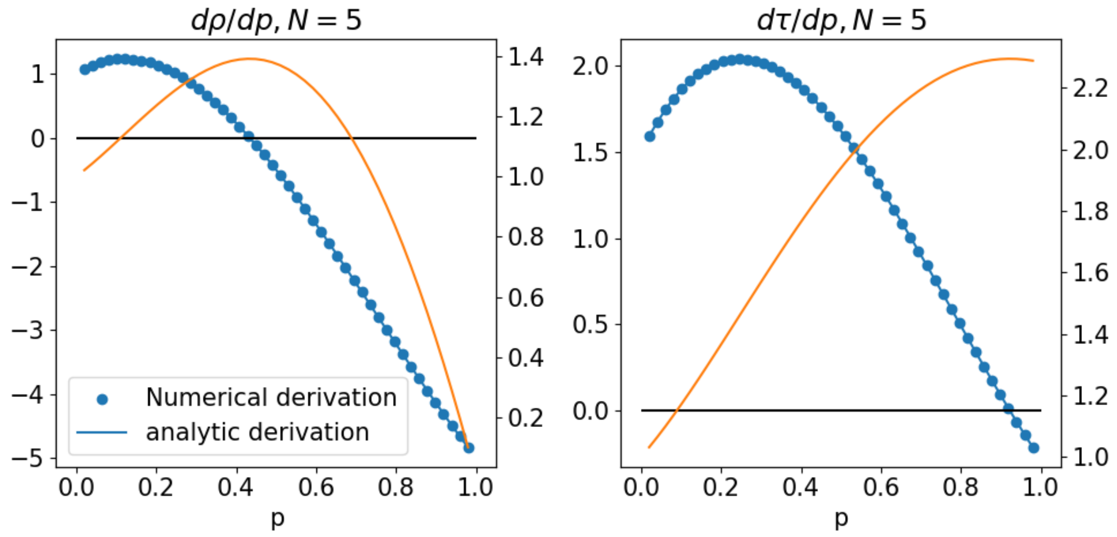

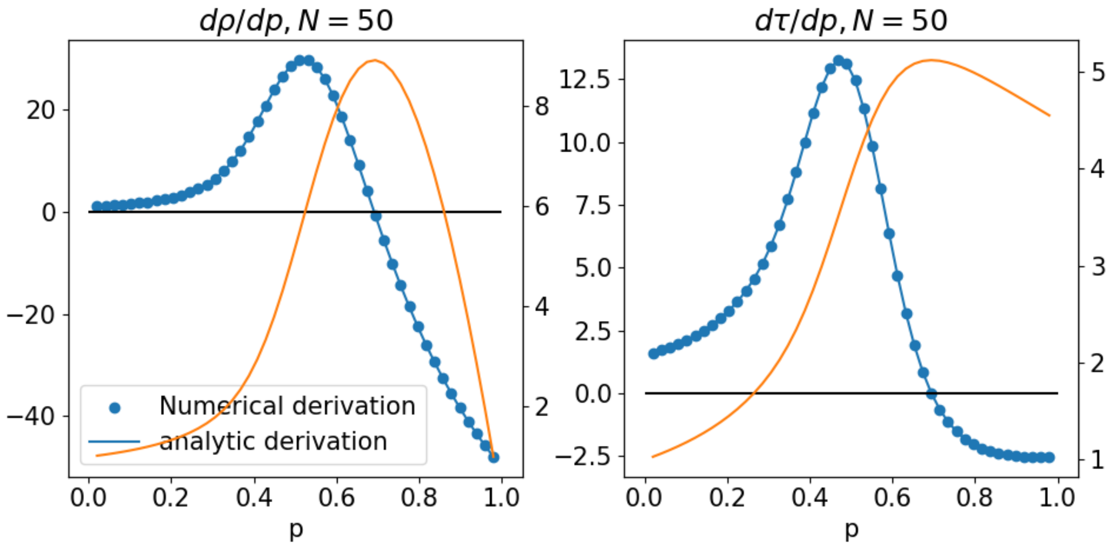

$d\rho/dp, N = 50$
$d\tau/dp, N = 50$
Numerical derivation
analytic derivation
p
p


```
[ ]:
```
