# Supplementary material for: Stability of ecologically scaffolded traits during evolutionary transitions in individuality
Source: Nat Commun. 2024 Aug 3;15:6566. doi: 10.1038/s41467-024-50625-1 (PMC11297203; doi:10.1038/s41467-024-50625-1)
Supplement: Supplementary file 4 — Supplementary Code 1 [file 41467_2024_50625_MOESM4_ESM.zip › code/results/notebook_exports/05_adaptive_dynamics.pdf]

Notebook 05_adaptive_dynamics.ipynb

Guilhem Doulcier

June 28, 2024

1 Adaptive dynamics

This notebook plots pairwise invasibility plots

[1]:
```
import matplotlib.pyplot as plt
import numpy as np
import pandas as pd
from scaffold import labels
from scaffold.meanfield.analytical import (grad_inv_fitness, inv_fitness,
                                            rho)
from scipy.optimize import fmin, fsolve

plt.rc('font', size=15)
```

2 Pairwise Invasibility Plots

[2]:
```
pspan = np.linspace(0,1,100)
X,Y = np.meshgrid(pspan,pspan)
Z = {}
Nlist =[5,25,100]
pstar = {}
for N in Nlist:
    Z[N] = np.zeros_like(X)
    for i,_ in enumerate(X.flat):
        Z[N].flat[i] = inv_fitness(X.flat[i], Y.flat[i], N)
    pstar[N] =  fsolve(lambda p: grad_inv_fitness(p, N), 0.5)
```

[3]:
```
def draw_pip(X,Y,Z, N, pstar, ax):
    ax.set(title=f"Pairwise invasibility plot (R={N})\n (Black = positive␣
 ↪invasion fitness)",
           xlabel=labels['resident'],
           ylabel=labels['mutant'],
           xlim=(X.min(),X.max()),
           ylim=(Y.min(),Y.max()),
           )
    lvls = [min(-10, Z.min()), 0, max(10, Z.max())]
    ax.contourf(X, Y, Z, levels=lvls, colors=['w','k'])
```

```
    ax.scatter(pstar, pstar, color='C0')
```

[4]:
```
fig, ax = plt.subplots(1,len(Nlist),figsize=(5*len(Nlist),5.2))
raw = []
for i, N in enumerate(Nlist):
    draw_pip(X,Y,Z[N], N, pstar[N], ax[i])
    raw.append({"R":N, "mutant_trait":X, "resident_trait":Y, "invasion_fitness":
 ↪Z[N], "ESS":pstar[N]})
    ax[i].set(title=f'R={N}')
fig.suptitle(f"Pairwise invasibility plots. (Black = positive invasion␣
 ↪fitness)")
plt.tight_layout()
plt.savefig("fig/supfig/s5_pip_list.svg")
plt.savefig("fig/supfig/s5_pip_list.pdf")
pd.json_normalize(pd.DataFrame(raw)).to_csv("source_data/s5_pip_list.csv")
```

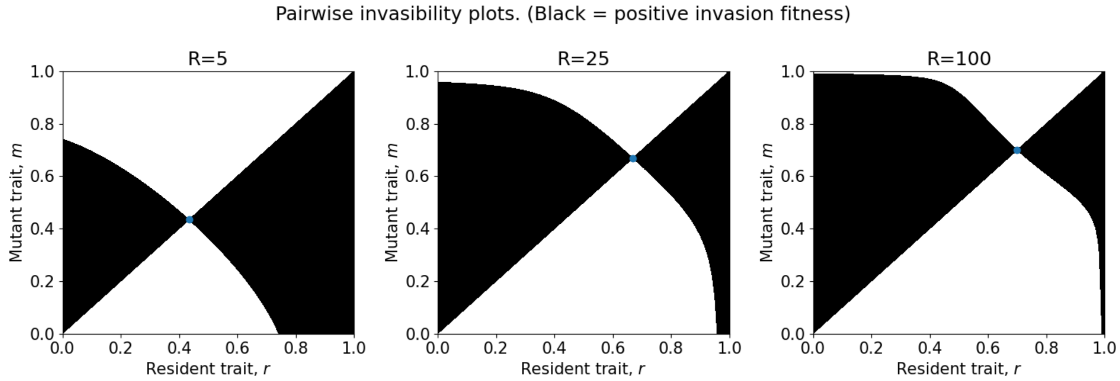

[5]:
```
plt.rc('font', size=15)
fig, ax = plt.subplots(1,1,figsize=(5,5))
draw_pip(X,Y,Z[25], 25, pstar[25], ax)
```

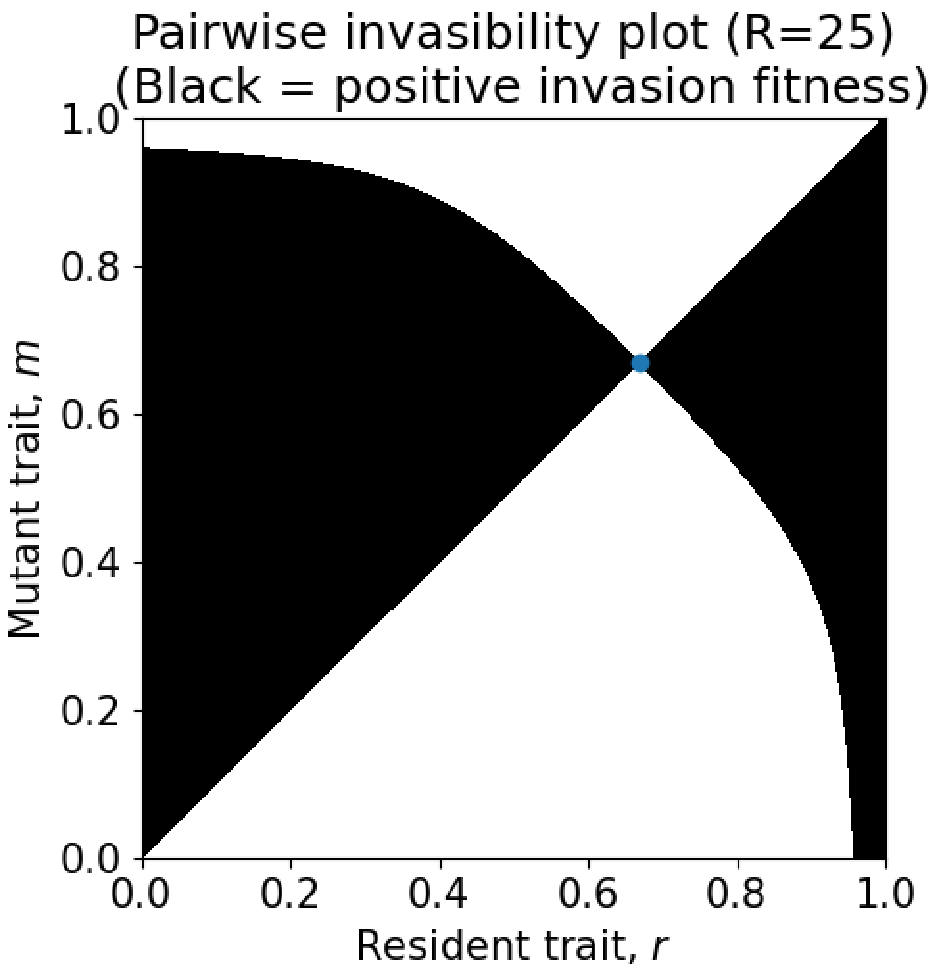

3 Checking the computation of the ESS

[6]:

```
Rspan = np.arange(2,100)
# two ways of computing it:
allpstar = [fsolve(lambda p: grad_inv_fitness(p, R), 0.5) for R in Rspan]
allpstar2 = [fmin(lambda p: -rho(p,R), 0.5, disp=0) for R in Rspan]

plt.scatter(allpstar, allpstar2)
plt.plot(allpstar, allpstar, color='k')
plt.gca().set(xlabel= 'ESS computed by roots of inv grad',
              ylabel= 'ESS computed by maxing rho')
```

[6]:

```
[Text(0.5, 0, 'ESS computed by roots of inv grad'),
 Text(0, 0.5, 'ESS computed by maxing rho')]
```

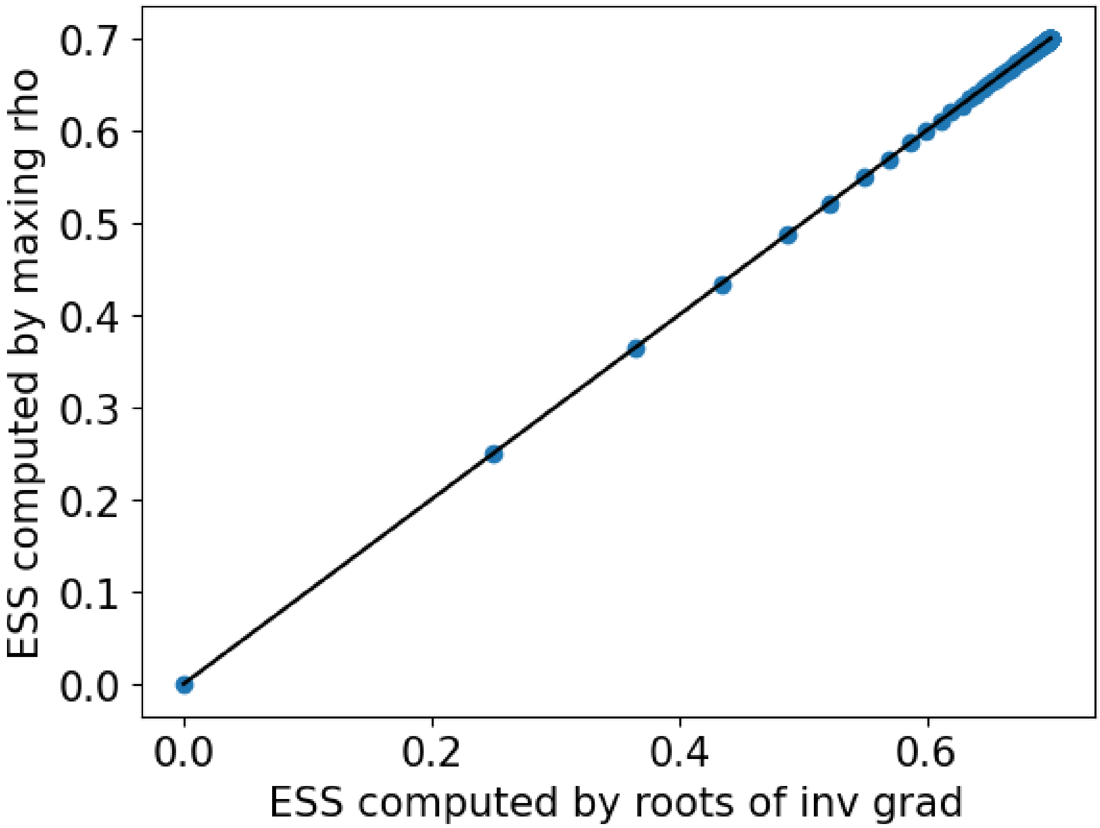
ESS computed by maxing rho
ESS computed by roots of inv grad
0.0
0.1
0.2
0.3
0.4
0.5
0.6
0.7
0.0
0.2
0.4
0.6

```
[ ]:
```
