# Supplementary material for: Stability of ecologically scaffolded traits during evolutionary transitions in individuality
Source: Nat Commun. 2024 Aug 3;15:6566. doi: 10.1038/s41467-024-50625-1 (PMC11297203; doi:10.1038/s41467-024-50625-1)
Supplement: Supplementary file 4 — Supplementary Code 1 [file 41467_2024_50625_MOESM4_ESM.zip › code/results/notebook_exports/06_bifurcation_diagram.pdf]

Notebook 06_bifurcation_diagram.ipynb

Guilhem Doulcier

June 28, 2024

This notebook produces figure 4c (rho as a function of theta) and s7 (bifurcation diagram on R)

[1]:
```
%load_ext autoreload
%autoreload 2
from functools import partial

import matplotlib.pyplot as plt
import numpy as np
import pandas as pd
import scipy.optimize

from scaffold.meanfield.general_rho import *
import scaffold.ecology
import scaffold.meanfield.analytical as analytical
from scaffold import labels
plt.rc('font', size=15)
```

[2]:
```
def plot_max(x,y, ax, **kargs):
    l = ax.plot(x, y, **kargs)
    diff = np.diff(y)
    locmax = np.where(diff[1:]*diff[:-1]<0)[0]
    ax.scatter(x[locmax], y[locmax], color=l[0].get_color())
    for u in locmax:
        ax.text(x[u], y[u], f"{x[u]:2.2}", color=l[0].get_color())
        ax.vlines(x[u],0, y[u], color=l[0].get_color(), ls='--')
    return ax, locmax, l[0].get_color()
```

0.1 Rho as a function of theta

[3]:
```
def rho_function_of_theta(proba, pmin=0, pmax=1, Rspan=[100,20], pplus=None,␣
↪showmax=True, ax=None):
    if ax is None:
        fig, ax = plt.subplots(1,1, figsize=(7.5,7.5))
    if pplus is None:
        pplus = [pmin,pmax]
    elif pplus[0] == 'lin':
        pplus = np.linspace(pmin,pmax,pplus[1])
```

```
    pspan = np.linspace(pmin,pmax,200)
    raw = pd.DataFrame({'trait':pspan})
    trait_max = []
    for i,R in enumerate(Rspan):
        try:
            mr = np.array([rho_from_matrix(generate_matrix(partial(proba, p=p),␣
↪R))for p in pspan])
        except ValueError:
            print(f"Value error: i={i} R={R}")
        else:
            if showmax:
                _, locmax,color = plot_max(pspan, mr, ax, label=f'R={R}')
                for u in locmax:
                    trait_max.append(pspan[u])
            else:
                ax.plot(pspan,mr,label=f'R={R}')
                raw[f'rho_R{R}'] = mr

        cst = [analytical.rho(p,R) for p in np.linspace(0,1,200)]
        amax = np.linspace(0,1,200)[np.argmax(cst)]

    x = np.arange(np.max(Rspan))

    ax.hlines(1,pmin,pmax, color='k')
    ax.legend()
    ax.set(xlabel=labels['trait'], ylabel=labels['rho'])
    return fig,ax,raw
```

[4]:
```
threshold = lambda pop,p: scaffold.ecology.threshold(trait=(1-p), pop=pop)
```

[5]:
```
fig, ax, raw = rho_function_of_theta(threshold, 0, .97, Rspan=[100,20],)
plt.savefig("fig/4c_rho_function_of_theta_density_dep_model.svg")
plt.savefig("fig/4c_rho_function_of_theta_density_dep_model.pdf")
raw.to_csv('source_data/4c_rho_function_of_theta_density_dep_model.csv')
```

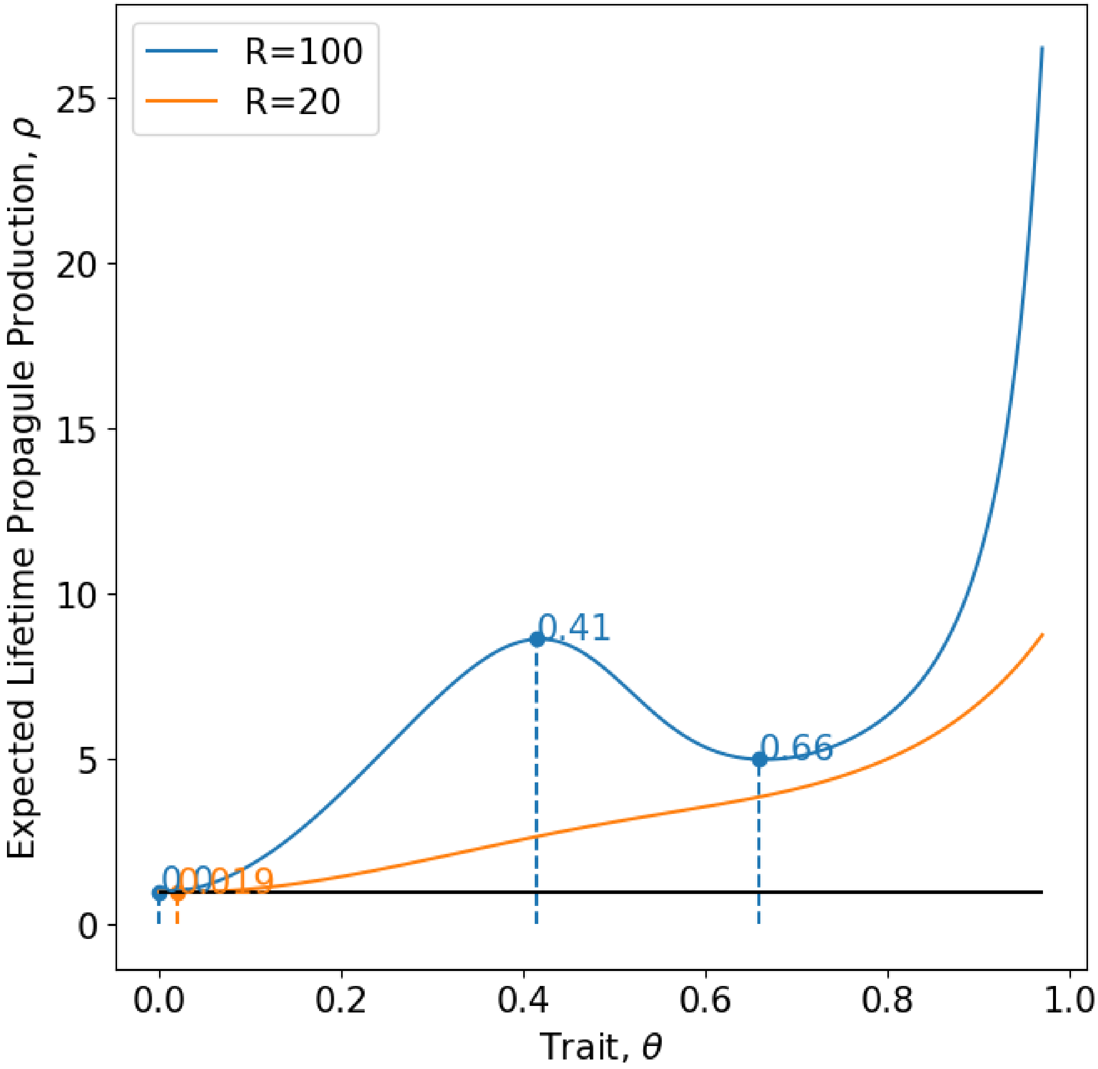

[6]:
```python
rho_function_of_theta(threshold, 0, 1, Rspan=np.arange(38,42)[::
    2],showmax=False)
```

[6]:
```
(<Figure size 750x750 with 1 Axes>,
 <Axes: xlabel='Trait, $\\theta$', ylabel='Expected Lifetime Propagule
Production, $\\rho$'>,
         trait     rho_R38     rho_R40
 0    0.000000    1.000000    1.000000
 1    0.005025    0.995930    0.995980
 2    0.010050    0.993752    0.993951
 3    0.015075    0.993438    0.993885
 4    0.020101    0.994961    0.995752
 ..        ...         ...         ...
 195  0.979899   15.960548   16.645896
```

```
196  0.984925  16.647728  17.400365
197  0.989950  17.380948  18.207936
198  0.994975  18.163757  19.072917
199  1.000000  19.000000  20.000000

[200 rows x 3 columns])
```

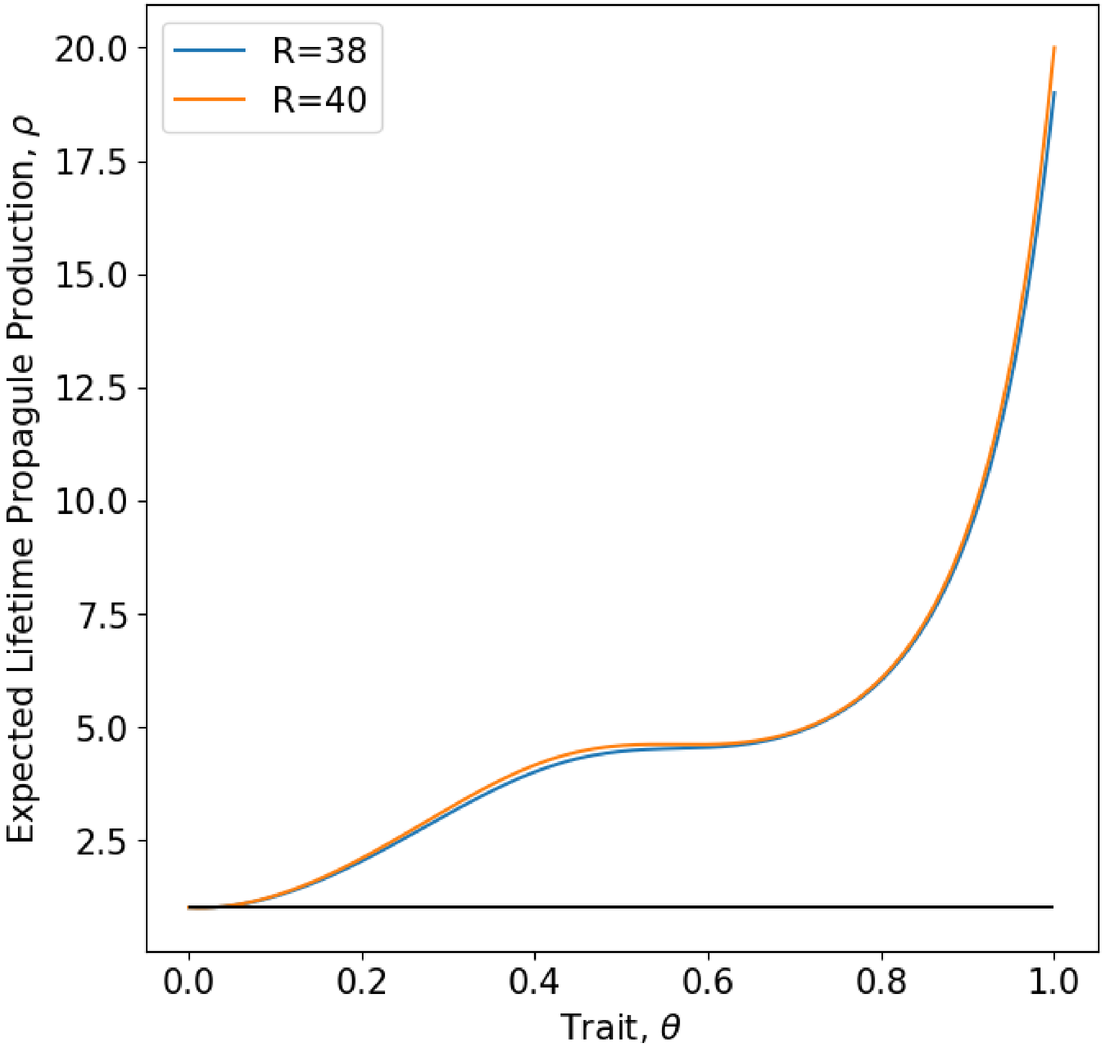

1 Bifurcation Diagram

1.1 Stable branch of the bifurcation diagram

[7]:
```
def to_optimize(p,R):
    try:
        return -rho_from_matrix(generate_matrix(lambda pop: scaffold.ecology.
 ↪threshold(trait=1-p, pop=pop), R)),
    except ValueError as ex:
        return 0
```

[8]:
```
start_R = 50
start_theta_max = 0.4
theta_max_left = []
theta_max_right = []
Rvalues_left = []
Rvalues_right = []

print("Natural parameter continuation")
print('left')
for R in np.arange(start_R,10, -1):
    try:
        start = start_theta_max if not len(theta_max_left) else␣
 ↪theta_max_left[-1]
        a = scipy.optimize.fmin(partial(to_optimize,R=R),
                                start,
                                disp=0, full_output=1,
                                xtol=1e-9, ftol=1e-9
                       )
        print(R, start, a)
        theta_max_left.append(a[0][0])
        Rvalues_left.append(R)
    except Exception as ex:
        print(ex)

print('right')
for R in np.arange(start_R,2*start_R, 1):
    try:
        start = start_theta_max if not len(theta_max_right) else␣
 ↪theta_max_right[-1]
        a = scipy.optimize.fmin(partial(to_optimize,R=R),
                                start,
                                disp=0, full_output=1,
                                xtol=1e-9, ftol=1e-9
                       )
        print(R, start, a)
        theta_max_right.append(a[0][0])
```

```
        Rvalues_right.append(R)
    except Exception as ex:
        print(ex)
```

```
Natural parameter continuation
left
50 0.4 (array([0.4775334]), np.float64(-5.190214762133763), 28, 58, 0)
49 0.4775334024429323 (array([0.48103947]), np.float64(-5.12742611737816), 26,
54, 0)
48 0.48103946634965183 (array([0.48483931]), np.float64(-5.064675514632874), 26,
54, 0)
47 0.48483931094632465 (array([0.48900362]), np.float64(-5.003232853047171), 27,
57, 0)
46 0.48900361577945234 (array([0.49360694]), np.float64(-4.941957751248841), 26,
53, 0)
45 0.4936069395495817 (array([0.49871235]), np.float64(-4.882235605193225), 26,
54, 0)
44 0.4987123530464432 (array([0.50453753]), np.float64(-4.822924394054691), 26,
54, 0)
43 0.5045375308356077 (array([0.51116275]), np.float64(-4.765530073830828), 26,
54, 0)
42 0.511162750787224 (array([0.51921047]), np.float64(-4.709056966471056), 26,
55, 0)
41 0.5192104700588741 (array([0.52910172]), np.float64(-4.655201822682676), 26,
55, 0)
40 0.5291017160921982 (array([0.54434278]), np.float64(-4.603804624642104), 26,
54, 0)
39 0.5443427777932306 (array([1.]), np.float64(-18.99999999996969), 57, 114, 0)
38 0.9999999999998217 (array([1.]), np.float64(-18.999999999969333), 35, 70, 0)
37 0.9999999999998217 (array([1.]), np.float64(-17.9999999999729), 34, 68, 0)
36 0.9999999999998217 (array([1.]), np.float64(-17.99999999997254), 34, 68, 0)
35 0.9999999999998217 (array([1.]), np.float64(-16.99999999997593), 34, 68, 0)
34 0.9999999999998217 (array([1.]), np.float64(-16.999999999975575), 34, 68, 0)
33 0.9999999999998217 (array([1.]), np.float64(-15.999999999978781), 34, 68, 0)
32 0.9999999999998217 (array([1.]), np.float64(-15.999999999978426), 34, 68, 0)
31 0.9999999999998217 (array([1.]), np.float64(-14.999999999981457), 34, 68, 0)
30 0.9999999999998217 (array([1.]), np.float64(-14.9999999999811), 34, 68, 0)
29 0.9999999999998217 (array([1.]), np.float64(-13.999999999983952), 34, 68, 0)
28 0.9999999999998217 (array([1.]), np.float64(-13.999999999983595), 34, 68, 0)
27 0.9999999999998217 (array([1.]), np.float64(-12.99999999998627), 33, 66, 0)
26 0.9999999999998217 (array([1.]), np.float64(-12.999999999985913), 33, 66, 0)
25 0.9999999999998217 (array([1.]), np.float64(-11.999999999988411), 33, 66, 0)
24 0.9999999999998217 (array([1.]), np.float64(-11.999999999988054), 33, 66, 0)
23 0.9999999999998217 (array([1.]), np.float64(-10.99999999999037), 33, 66, 0)
22 0.9999999999998217 (array([1.]), np.float64(-10.999999999990015), 33, 66, 0)
21 0.9999999999998217 (array([1.]), np.float64(-9.999999999992156), 33, 66, 0)
```

```
20 0.9999999999998217 (array([1.]), np.float64(-9.999999999991799), 33, 66, 0)
19 0.9999999999998217 (array([1.]), np.float64(-8.99999999999376), 32, 64, 0)
18 0.9999999999998217 (array([1.]), np.float64(-8.999999999993403), 32, 64, 0)
17 0.9999999999998217 (array([1.]), np.float64(-7.999999999995186), 32, 64, 0)
16 0.9999999999998217 (array([1.]), np.float64(-7.999999999994829), 32, 64, 0)
15 0.9999999999998217 (array([1.]), np.float64(-6.999999999996434), 31, 62, 0)
14 0.9999999999998217 (array([1.]), np.float64(-6.999999999996077), 32, 64, 0)
13 0.9999999999998217 (array([1.]), np.float64(-5.999999999997503), 31, 62, 0)
12 0.9999999999998217 (array([1.]), np.float64(-5.999999999997147), 31, 62, 0)
11 0.9999999999998217 (array([1.]), np.float64(-4.999999999998396), 30, 60, 0)
right
50 0.4 (array([0.4775334]), np.float64(-5.190214762133763), 28, 58, 0)
51 0.4775334024429323 (array([0.47431186]), np.float64(-5.254127190639609), 26,
54, 0)
52 0.4743118567715874 (array([0.47129845]), np.float64(-5.3180061552693765), 26,
54, 0)
53 0.47129845186105934 (array([0.46851654]), np.float64(-5.3828624704606485),
26, 54, 0)
54 0.46851653739160914 (array([0.46588807]), np.float64(-5.44764614621026), 26,
56, 0)
55 0.46588806695246254 (array([0.46345289]), np.float64(-5.5132869115321395),
26, 53, 0)
56 0.46345289454749955 (array([0.46113429]), np.float64(-5.5788348015181), 27,
57, 0)
57 0.4611342897609435 (array([0.45897993]), np.float64(-5.645138988399327), 26,
55, 0)
58 0.45897992679159383 (array([0.45691629]), np.float64(-5.711341460124831), 26,
56, 0)
59 0.45691629184609994 (array([0.45499397]), np.float64(-5.778214581587357), 26,
55, 0)
60 0.45499397004957476 (array([0.45314372]), np.float64(-5.844984072803664), 26,
54, 0)
61 0.45314371534375697 (array([0.4514162]), np.float64(-5.912350707635539), 26,
54, 0)
62 0.4514162004344954 (array([0.44974694]), np.float64(-5.979615978933849), 26,
53, 0)
63 0.4497469358320699 (array([0.44818508]), np.float64(-6.0474148254741795), 26,
54, 0)
64 0.44818507654045214 (array([0.44667104]), np.float64(-6.115117035367956), 26,
54, 0)
65 0.4466710435229587 (array([0.44525158]), np.float64(-6.18329751041027), 26,
54, 0)
66 0.44525157665851023 (array([0.44387191]), np.float64(-6.251387439976056), 26,
53, 0)
```

```
67 0.44387191432422746 (array([0.44257593]), np.float64(-6.319907265982142), 27,
57, 0)
68 0.442575934552518 (array([0.44131352]), np.float64(-6.388343300000079), 26,
53, 0)
69 0.4413135179231346 (array([0.44012549]), np.float64(-6.457166748560711), 26,
53, 0)
70 0.4401254918181321 (array([0.43896607]), np.float64(-6.525913372048784), 26,
54, 0)
71 0.43896607041839836 (array([0.43787304]), np.float64(-6.59500995682453), 26,
54, 0)
72 0.43787304144516725 (array([0.43680467]), np.float64(-6.664036612985932), 26,
53, 0)
73 0.43680466533682716 (array([0.43579574]), np.float64(-6.733380098677987), 26,
54, 0)
74 0.43579574468698473 (array([0.43480828]), np.float64(-6.802660306545958), 28,
57, 0)
75 0.4348082762451192 (array([0.43387421]), np.float64(-6.872227945114166), 26,
55, 0)
76 0.4338742104909433 (array([0.43295898]), np.float64(-6.941738607748393), 26,
54, 0)
77 0.4329589757864781 (array([0.43209185]), np.float64(-7.011510541253866), 26,
54, 0)
78 0.43209185092278213 (array([0.43124141]), np.float64(-7.081231396309691), 27,
58, 0)
79 0.43124141203075383 (array([0.43043442]), np.float64(-7.151190184042006), 26,
55, 0)
80 0.4304344249461701 (array([0.4296423]), np.float64(-7.221103362415734), 26,
55, 0)
81 0.4296423031599901 (array([0.42888956]), np.float64(-7.291233602141475), 27,
57, 0)
82 0.4288895565906221 (array([0.42815014]), np.float64(-7.3613232698145), 26,
54, 0)
83 0.4281501361778668 (array([0.42744646]), np.float64(-7.431611291278591), 26,
53, 0)
84 0.4274464575079399 (array([0.42675482]), np.float64(-7.501863356012681), 26,
53, 0)
85 0.4267548220182743 (array([0.4260957]), np.float64(-7.572296970586067), 26,
54, 0)
86 0.4260956994528817 (array([0.42544754]), np.float64(-7.642698839737136), 27,
56, 0)
```

```
87 0.4254475366682485 (array([0.424829]), np.float64(-7.713267134181047), 26,
54, 0)

88 0.4248289961022721 (array([0.42422047]), np.float64(-7.7838075132149225), 26,
55, 0)

89 0.42422047318030864 (array([0.42363899]), np.float64(-7.854500678460141), 26,
55, 0)

90 0.42363898734990424 (array([0.42306671]), np.float64(-7.925169402174862), 27,
55, 0)

91 0.42306671468024293 (array([0.42251919]), np.float64(-7.995978590150219), 26,
54, 0)

92 0.4225191886209426 (array([0.42198013]), np.float64(-8.066766480400208), 27,
58, 0)

93 0.421980134161681 (array([0.42146378]), np.float64(-8.137683683521683), 26,
53, 0)

94 0.4214637779280087 (array([0.42095526]), np.float64(-8.208582428581803), 26,
54, 0)

95 0.42095525954980556 (array([0.42046758]), np.float64(-8.279600377692965), 26,
53, 0)

96 0.4204675804913587 (array([0.41998718]), np.float64(-8.350602429417114), 27,
55, 0)

97 0.41998718047365563 (array([0.41952594]), np.float64(-8.421714506862857), 26,
53, 0)

98 0.4195259446344945 (array([0.4190715]), np.float64(-8.49281299257116), 27,
57, 0)

99 0.41907149613644823 (array([0.4186347]), np.float64(-8.564013157767798), 27,
55, 0)
```

1.2 Unstable branch of the bifurcation diagram

[9]:
```python
def to_optimize2(p,R):
    try:
        return rho_from_matrix(generate_matrix(lambda pop: scaffold.ecology.
 ↪threshold(trait=1-p, pop=pop), R)),
    except ValueError as ex:
        return 0


start_R = 50
start_theta_max = 0.7
theta_min_left = []
theta_min_right = []
```

```
Rvalues_left = []
Rvalues_right = []

for R in np.arange(start_R,10, -1):
    try:
        start = start_theta_max if not len(theta_min_left) else␣
↪theta_min_left[-1]
        a = scipy.optimize.fmin(partial(to_optimize2,R=R),
                                start,
                                disp=0, full_output=1,
                                xtol=1e-9, ftol=1e-9
                          )
        print(R, start, a)
        theta_min_left.append(a[0][0])
        Rvalues_left.append(R)
    except Exception as ex:
        print(ex)
        raise ex

for R in np.arange(start_R,2*start_R, 1):
    try:
        start = start_theta_max if not len(theta_min_right) else␣
↪theta_min_right[-1]
        a = scipy.optimize.fmin(partial(to_optimize2,R=R),
                                start,
                                disp=0, full_output=1,
                                xtol=1e-9, ftol=1e-9
                          )
        print(R, start, a)
        theta_min_right.append(a[0][0])
        Rvalues_right.append(R)
    except Exception as ex:
        pass
```

```
50 0.7 (array([0.63658021]), np.float64(4.818458669878452), 30, 62, 0)
49 0.6365802136063572 (array([0.63433783]), np.float64(4.8039068665920315), 26,
54, 0)

48 0.6343378329917899 (array([0.6316569]), np.float64(4.789178973329356), 26,
54, 0)
47 0.6316568978485757 (array([0.62887059]), np.float64(4.77204948032179), 27,
56, 0)

46 0.6288705858468462 (array([0.62546271]), np.float64(4.754503671478035), 26,
54, 0)
45 0.6254627074111068 (array([0.62186826]), np.float64(4.73420115791884), 26,
54, 0)

44 0.6218682574986283 (array([0.61732434]), np.float64(4.713081153720948), 26,
```

55, 0)
43 0.6173243446218735 (array([0.61236599]), np.float64(4.688742928143722), 26, 54, 0)

42 0.6123659890066425 (array([0.60569637]), np.float64(4.662854197476654), 26, 54, 0)
41 0.6056963700918484 (array([0.59765623]), np.float64(4.6329747792981415), 27, 56, 0)
40 0.5976562342755491 (array([0.58390337]), np.float64(4.599695889502074), 27, 55, 0)

39 0.5839033695794853 (array([-0.05839034]), np.float64(0.0), 33, 93, 0)
38 -0.05839033695794926 (array([-0.05839034]), np.float64(0.0), 23, 68, 0)
37 -0.05839033695794926 (array([-0.05839034]), np.float64(0.0), 23, 68, 0)
36 -0.05839033695794926 (array([-0.05839034]), np.float64(0.0), 23, 68, 0)
35 -0.05839033695794926 (array([-0.05839034]), np.float64(0.0), 23, 68, 0)
34 -0.05839033695794926 (array([-0.05839034]), np.float64(0.0), 23, 68, 0)
33 -0.05839033695794926 (array([-0.05839034]), np.float64(0.0), 23, 68, 0)

32 -0.05839033695794926 (array([-0.05839034]), np.float64(0.0), 23, 68, 0)
31 -0.05839033695794926 (array([-0.05839034]), np.float64(0.0), 23, 68, 0)
30 -0.05839033695794926 (array([-0.05839034]), np.float64(0.0), 23, 68, 0)
29 -0.05839033695794926 (array([-0.05839034]), np.float64(0.0), 23, 68, 0)
28 -0.05839033695794926 (array([-0.05839034]), np.float64(0.0), 23, 68, 0)
27 -0.05839033695794926 (array([-0.05839034]), np.float64(0.0), 23, 68, 0)
26 -0.05839033695794926 (array([-0.05839034]), np.float64(0.0), 23, 68, 0)
25 -0.05839033695794926 (array([-0.05839034]), np.float64(0.0), 23, 68, 0)
24 -0.05839033695794926 (array([-0.05839034]), np.float64(0.0), 23, 68, 0)
23 -0.05839033695794926 (array([-0.05839034]), np.float64(0.0), 23, 68, 0)
22 -0.05839033695794926 (array([-0.05839034]), np.float64(0.0), 23, 68, 0)
21 -0.05839033695794926 (array([-0.05839034]), np.float64(0.0), 23, 68, 0)

20 -0.05839033695794926 (array([-0.05839034]), np.float64(0.0), 23, 68, 0)
19 -0.05839033695794926 (array([-0.05839034]), np.float64(0.0), 23, 68, 0)
18 -0.05839033695794926 (array([-0.05839034]), np.float64(0.0), 23, 68, 0)
17 -0.05839033695794926 (array([-0.05839034]), np.float64(0.0), 23, 68, 0)
16 -0.05839033695794926 (array([-0.05839034]), np.float64(0.0), 23, 68, 0)
15 -0.05839033695794926 (array([-0.05839034]), np.float64(0.0), 23, 68, 0)
14 -0.05839033695794926 (array([-0.05839034]), np.float64(0.0), 23, 68, 0)
13 -0.05839033695794926 (array([-0.05839034]), np.float64(0.0), 23, 68, 0)
12 -0.05839033695794926 (array([-0.05839034]), np.float64(0.0), 23, 68, 0)
11 -0.05839033695794926 (array([-0.05839034]), np.float64(0.0), 23, 68, 0)

50 0.7 (array([0.63658021]), np.float64(4.818458669878452), 30, 62, 0)

51 0.6365802136063572 (array([0.63875155]), np.float64(4.830906668045086), 27, 56, 0)
52 0.6387515513874205 (array([0.64060025]), np.float64(4.843326698520007), 26, 53, 0)

53 0.64060025016847 (array([0.64239396]), np.float64(4.853900827959318), 26, 54,

0)

54 0.6423939612318648 (array([0.64394406]), np.float64(4.864538176543954), 26, 53, 0)
55 0.6439440646679131 (array([0.64544728]), np.float64(4.8735557046428815), 27, 56, 0)

56 0.6454472845055963 (array([0.64676335]), np.float64(4.882691022726614), 26, 53, 0)

57 0.6467633508099015 (array([0.64803703]), np.float64(4.890405512634787), 26, 55, 0)
58 0.6480370306865522 (array([0.64916515]), np.float64(4.898268351690758), 26, 53, 0)

59 0.6491651517960075 (array([0.65025373]), np.float64(4.904885640559493), 26, 54, 0)
60 0.6502537285776412 (array([0.65122809]), np.float64(4.911665990374974), 26, 54, 0)

61 0.65122808502535 (array([0.65216502]), np.float64(4.917355082654475), 26, 55, 0)
62 0.6521650153852564 (array([0.65301171]), np.float64(4.923211532978368), 26, 54, 0)

63 0.6530117111361553 (array([0.65382278]), np.float64(4.928112393474921), 26, 54, 0)
64 0.6538227788387262 (array([0.65456224]), np.float64(4.933178169103054), 27, 56, 0)

65 0.6545622375421543 (array([0.65526777]), np.float64(4.9374075003609645), 26, 52, 0)
66 0.6552677653595615 (array([0.65591628]), np.float64(4.941795071607811), 26, 54, 0)

67 0.6559162834499965 (array([0.65653251]), np.float64(4.945450713777555), 26, 54, 0)
68 0.6565325091968511 (array([0.65710327]), np.float64(4.9492554049781905), 26, 52, 0)

69 0.6571032687574965 (array([0.65764342]), np.float64(4.9524197566743435), 26, 53, 0)
70 0.6576434214877034 (array([0.65814723]), np.float64(4.955722622396974), 26, 54, 0)

71 0.6581472254465374 (array([0.65862211]), np.float64(4.958465343788413), 26, 54, 0)
72 0.6586221115690243 (array([0.65906797]), np.float64(4.961335493424244), 26, 52, 0)

73 0.6590679683053229 (array([0.65948659]), np.float64(4.963715669825898), 26, 53, 0)

```
74 0.6594865871598484 (array([0.65988202]), np.float64(4.966212166639014), 27,
56, 0)

75 0.659882018512753 (array([0.66025188]), np.float64(4.968280058373388), 26,
53, 0)
76 0.6602518831877614 (array([0.66060324]), np.float64(4.9704534840542225), 26,
54, 0)

77 0.6606032442755294 (array([0.66093072]), np.float64(4.972251953806819), 26,
53, 0)
78 0.6609307184561082 (array([0.66124344]), np.float64(4.97414570615839), 26,
54, 0)

79 0.6612434431536416 (array([0.66153389]), np.float64(4.975711391545348), 26,
54, 0)
80 0.6615338867636713 (array([0.66181261]), np.float64(4.977362766724231), 27,
55, 0)

81 0.6618126090847862 (array([0.6620706]), np.float64(4.978727049326269), 27,
58, 0)
82 0.6620706010439965 (array([0.66231938]), np.float64(4.98016814849223), 27,
56, 0)

83 0.6623193804292713 (array([0.66254887]), np.float64(4.981357958784554), 26,
52, 0)

84 0.6625488689489594 (array([0.66277111]), np.float64(4.982616450534683), 26,
54, 0)

85 0.6627711084441712 (array([0.66297553]), np.float64(4.983654939465083), 26,
55, 0)

86 0.6629755270662927 (array([0.66317427]), np.float64(4.984754703072461), 26,
54, 0)

87 0.6631742749046337 (array([0.66335654]), np.float64(4.985661804552858), 26,
53, 0)

88 0.6633565407323454 (array([0.66353443]), np.float64(4.986623474171851), 26,
53, 0)

89 0.6635344292595351 (array([0.66369709]), np.float64(4.987416377819789), 27,
55, 0)

90 0.6636970943490508 (array([0.66385644]), np.float64(4.988257804046854), 26,
54, 0)

91 0.6638564397312665 (array([0.66400173]), np.float64(4.988951353717235), 27,
56, 0)

92 0.6640017348537632 (array([0.66414456]), np.float64(4.989687995924836), 26,
56, 0)

93 0.6641445624516638 (array([0.66427445]), np.float64(4.99029502661221), 26,
53, 0)
```

```
94 0.6642744523653608 (array([0.66440253]), np.float64(4.9909402871069135), 26,
55, 0)

95 0.6644025305279422 (array([0.66451874]), np.float64(4.99147191046758), 26,
53, 0)

96 0.6645187410616705 (array([0.66463366]), np.float64(4.992037419620383), 26,
52, 0)

97 0.6646336608836074 (array([0.66473768]), np.float64(4.992503266510033), 26,
53, 0)

98 0.6647376786540993 (array([0.66484084]), np.float64(4.992999126464119), 26,
54, 0)

99 0.6648408370696639 (array([0.664934]), np.float64(4.9934075534028475), 26,
54, 0)
```

2 Plot the full bifurcation diagram

[10]:

```
fig, ax = plt.subplots(1,1, figsize=(8,5))
plt.scatter(Rvalues_left, theta_max_left, marker='.', color='C0',␣
↪label='Stable')
plt.scatter(Rvalues_right, theta_max_right, marker='.', color='C0')
plt.scatter(Rvalues_right, [1]*len(Rvalues_right), marker='.',color='C0')
plt.scatter(Rvalues_left, [1]*len(Rvalues_left), marker='.',color='C0')
plt.scatter(Rvalues_right, theta_min_right, color='C7', marker='.',␣
↪label='Unstable')
plt.scatter(Rvalues_left, theta_min_left, color='C7', marker='.')
plt.vlines(39, 0,0.7, color='k', ls=":")
ax.legend(loc='lower left')
ax.set(xlabel=labels['R'],
       ylabel=labels['trait'],
       ylim=[0,1.05])

raw = {"R":np.concat([Rvalues_left, Rvalues_right]),
       "stable_branch_1":np.concat([theta_max_left, theta_max_right]),
       "unstable_branch":np.concat([theta_min_left, theta_min_right]),
       "stable_branch_2":np.concat([[1]*len(Rvalues_right),␣
↪[1]*len(Rvalues_left)])}

pd.DataFrame(raw).to_csv('source_data/s7_ESS_density_dep.csv')
fig.savefig("fig/supfig/s7_ESS_density_dep.svg", bbox_inches='tight')
```

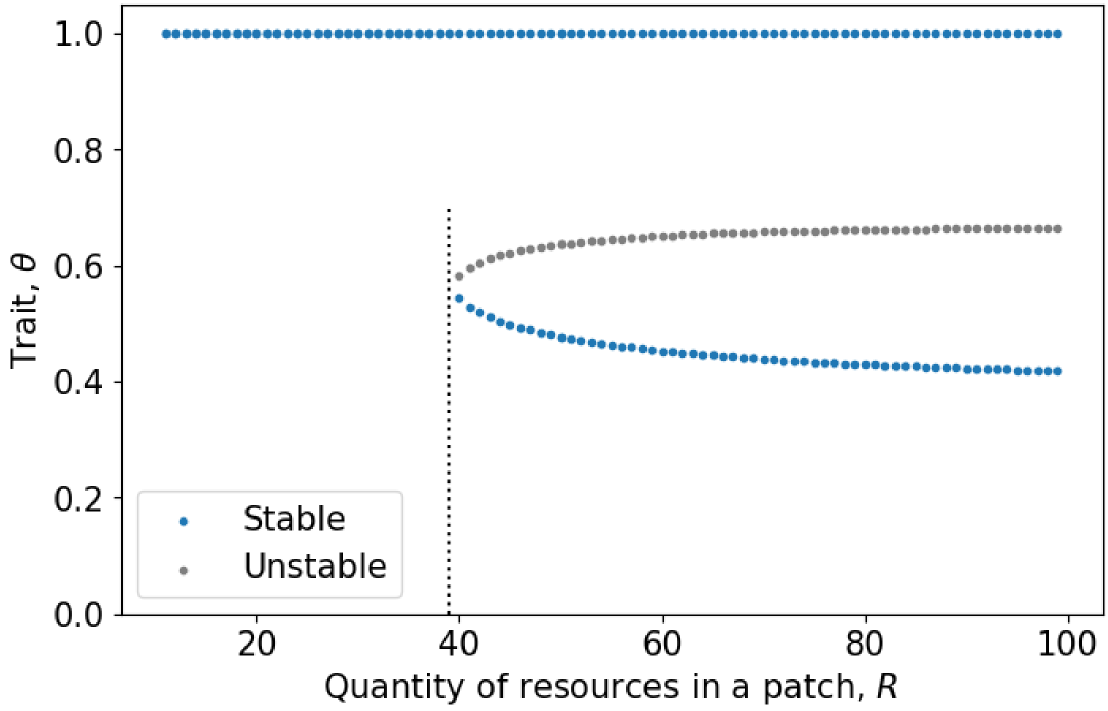
1.0
0.8
0.6
0.4
0.2
0.0
Trait, θ
20
40
60
80
100
Quantity of resources in a patch, R
Stable
Unstable

```
[ ]:
```
