# Supplementary material for: Stability of ecologically scaffolded traits during evolutionary transitions in individuality
Source: Nat Commun. 2024 Aug 3;15:6566. doi: 10.1038/s41467-024-50625-1 (PMC11297203; doi:10.1038/s41467-024-50625-1)
Supplement: Supplementary file 4 — Supplementary Code 1 [file 41467_2024_50625_MOESM4_ESM.zip › code/results/notebook_exports/07_occupancy.pdf]

Notebook 07_occupancy.ipynb

Guilhem Doulcier

June 28, 2024

This notebook parse the result of `07_occupancy.py` plots figures S1

[1]:
```
import matplotlib.pyplot as plt
import numpy as np
import pandas as pd
import scaffold.meanfield.analytical
from scaffold import labels
```

[2]:
```
data = pd.read_csv("output/occupancy.csv", index_col=0)
mean = data.groupby(['R','p']).mean()
std = data.groupby(['R','p']).std()
count = data.groupby(['R','p']).count()
count.columns = [x+'_count' for x in count.columns]
stats = pd.merge(mean, std, suffixes=['_mean','_std'], left_index=True,␣
↪right_index=True)
stats = pd.merge(stats, count, left_index=True, right_index=True)
```

[3]:
```
fig, ax = plt.subplots(1,1, figsize=(8,5))
pspan = np.linspace(0,1,200)

for R, df in stats.groupby('R'):
    df = df.reset_index()
    l = ax.plot(pspan,
                [scaffold.meanfield.analytical.occupancy(p,R,D=100,d=1) for p␣
↪in pspan],
                label=f'R = {R}')
    ax.errorbar(df.p, df.occupancy_mean, yerr=df.occupancy_std, fmt='.',␣
↪color=l[0].get_color())

ax.legend(title=f"line: analytical result\npoints: average of {stats.
↪occupancy_count.min()} simulations\nerror bar: standard deviations.")
ax.set(xlabel=labels['theta'], ylabel=labels['occupancy'])
fig.savefig("fig/supfig/s1_occupancy_sto.svg")
fig.savefig("fig/supfig/s1_occupancy_sto.pdf")
stats.to_csv('source_data/s1_occupancy_sto.csv')
```

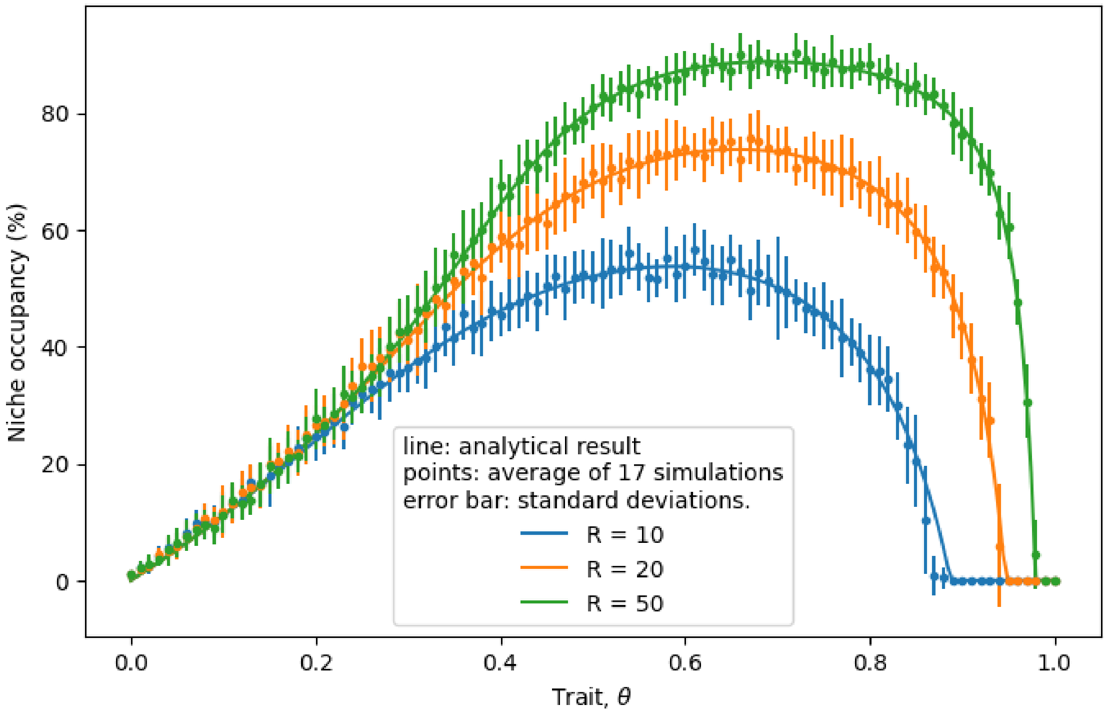

Niche occupancy (%)
80
60
40
20
0
0.0
0.2
0.4
0.6
0.8
1.0
Trait, θ
line: analytical result
points: average of 17 simulations
error bar: standard deviations.
R = 10
R = 20
R = 50
