# Supplementary material for: Stability of ecologically scaffolded traits during evolutionary transitions in individuality
Source: Nat Commun. 2024 Aug 3;15:6566. doi: 10.1038/s41467-024-50625-1 (PMC11297203; doi:10.1038/s41467-024-50625-1)
Supplement: Supplementary file 4 — Supplementary Code 1 [file 41467_2024_50625_MOESM4_ESM.zip › code/results/notebook_exports/08_collective_size.pdf]

Notebook 08_collective_size.ipynb

Guilhem Doulcier

June 28, 2024

In this notebook we look at the average and maximum collective sizes and produced figure S8 and S9

[1]:
```
import numpy as np
import os
import matplotlib.pyplot as plt
import pandas as pd
from functools import partial
from collections import namedtuple
from mpl_toolkits.axes_grid1 import make_axes_locatable
import scaffold.stochastic_single_patch as sp
import scaffold.stochastic_mixed as sm

dataset = {}
plt.rc('font', size=15)
os.makedirs("output/csize_01", exist_ok=True)
```

[2]:
```
setup = namedtuple("setup", ["name", "R0", "proba", "p1", "p2"])
exps = [setup("classic", 100, sp.classic, (0,1,100), None),
        setup("smooth", 100, sp.smooth, (0,1,31), (1,2,10)),
        setup("threshold", 100, sp.threshold, (0,1,31), (1,10,9)),
       ]
```

[3]:
```
for k in exps:
    key = k.name+"_R"+str(k.R0)+(str(k.p2) if k.p2 else '')
    f = f"output/csize_01/{key}.csv"

    if key not in dataset:
        print(f"NEW: {key}")
        if os.path.exists(f):
            dataset[key] = pd.read_csv(f)
        else:
            dataset[key] = sp.get_data(k)
            dataset[key].to_csv(f)

print(', '.join(dataset.keys()))
```

```
NEW: classic_R100
NEW: smooth_R100(1, 2, 10)
```

NEW: threshold_R100(1, 10, 9)
classic_R100, smooth_R100(1, 2, 10), threshold_R100(1, 10, 9)

[4]:
```
fig, ax = plt.subplots(1,3, figsize=(22,5))
data = {"Main model": (dataset['classic_R100'], sp.classic),
        "Smoothed model $k=2$": (dataset['smooth_R100(1, 2, 10)'].
 ↪query('p2==2'), partial(sp.smooth, p2=1)),
        "Threshold model $k=3$": (dataset['threshold_R100(1, 10, 9)'].
 ↪query('p2==3.25'), partial(sp.threshold, p2=3.25)),
 }
for label, (dd, p ) in data.items():
    ax[0].scatter(dd.p1, dd.propagules_mean, label=label)
    ax[1].scatter(dd.p1, dd.mx_mean)
    ax[1].axhline(2,color='k')
    ax[0].grid()
    ax[1].grid()
    ax[0].set(xlabel=r'$\theta$', ylabel='Average number of Propagules')
    ax[1].set(xlabel=r'$\theta$', ylabel='Average Maximum collective size')
    x = np.arange(1,7)
    ax[2].plot(x, [p(xx, p1=1) for xx in x], marker='o', ls=':')
    ax[2].set(xlabel="Number of propagules",
              ylabel=r"Probability to disperse for $\theta=1$")
ax[0].legend(loc='upper center')

for i in range(3):
    ax[i].text(0.06,0.95,'abc'[i], horizontalalignment='right',
         verticalalignment='top',
         bbox=dict(facecolor='none', edgecolor='black', boxstyle='round,pad=0.
 ↪2'),
         transform=ax[i].transAxes)


dataset['classic_R100'].to_csv('source_data/s9_main_model.csv')
dataset['smooth_R100(1, 2, 10)'].query('p2==2').to_csv('source_data/
 ↪s9_smoothed_k2.csv')
dataset['threshold_R100(1, 10, 9)'].query('p2==3.25').to_csv('source_data/
 ↪s9_threshold_k3.csv')
plt.savefig("fig/supfig/s9_size_select.svg", bbox_inches='tight')
plt.savefig("fig/supfig/s9_size_select.pdf", bbox_inches='tight')
```

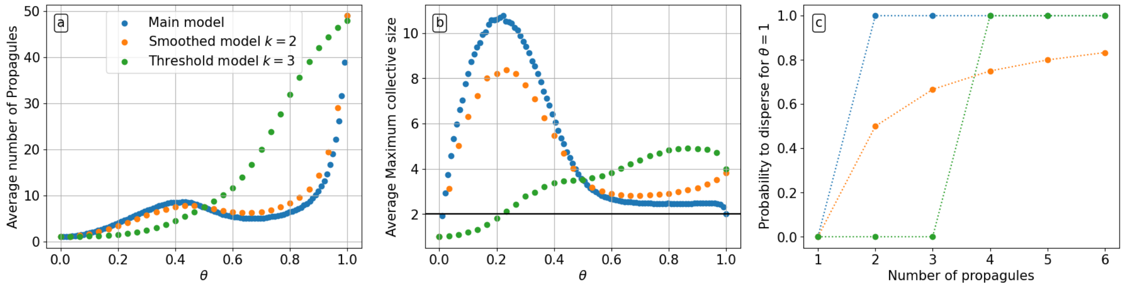

1 Visualisation of a trajectory

[5]:
```
np.random.seed(3)
data_cl = sm.stochastic_no_mut(partial(sm.classic, p=1), R0=12, D=10,␣
  ↪events=500)
data_sm = sm.stochastic_no_mut(partial(sm.smooth, p=1), R0=12, D=10, events=500)

plt.gca().set(xlabel="time", ylabel='Population')
plt.plot(data_cl[1], data_cl[3], label="Classic")
plt.plot(data_sm[1], data_sm[3], label="Smooth")

plt.legend()
```

[5]: <matplotlib.legend.Legend at 0x7f1dcdfcf2d0>

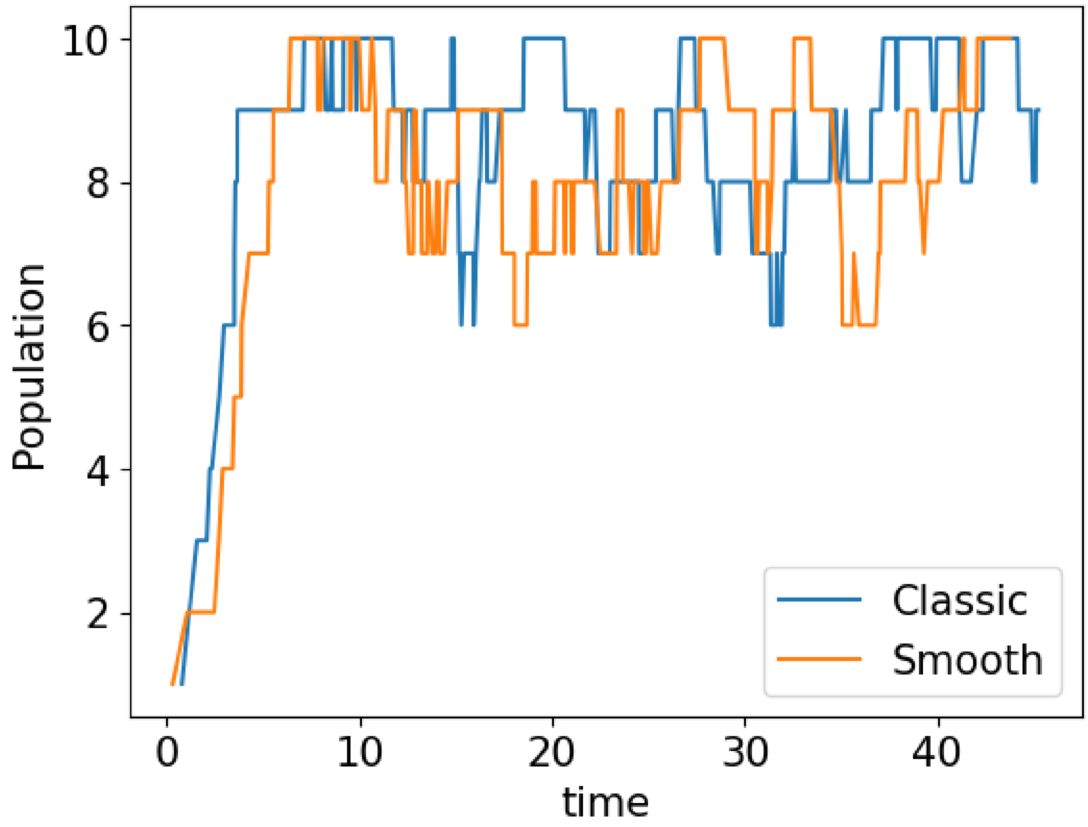


```
[6]: def plot_dyn(events, ax, scale=0.4/5, tmax=25, D=10):
        scale_r = 0.4/events.R_focal.max()
        R0 = events.R_focal.max()
        for k in events.focal.unique():
            df = events.loc[np.logical_or(events.focal==k, events.target==k)]
            p = np.array([0]+[x.pop_focal if x.focal==k else x.pop_target for _,x␣
     ↪in df.iterrows()])
            R = np.array([R0]+[x.R_focal if x.focal==k else x.R_target for _,x in␣
     ↪df.iterrows()])
            ax.fill_betweenx( [0]+list(df.time),scale_r*R+k ,k-scale_r*R, alpha=0.
     ↪4, step='post', ec="C5", fc='C5')
            ax.fill_betweenx( [0]+list(df.time),scale*p+k ,k-scale*p, color="C1",␣
     ↪alpha=0.5, ec='none', step='post')
        for k in np.arange(D-1):
            if k not in events.focal.unique():
                p = np.array([0,0])
                R = np.array([R0,R0])
                ax.fill_betweenx( [0,tmax],scale_r*R+k ,k-scale_r*R, alpha=0.4,␣
     ↪step='post', ec="C5", fc='C5')
```

```
            ax.fill_betweenx( [0,tmax],scale*p+k ,k-scale*p, color="C1",␣
↪alpha=0.5, ec='none', step='post')

    prop = events[events.birth==False]
    if prop.shape[0]:
        for _, row in prop[prop.target!=np.nan].iterrows():
            ax.arrow(row.focal, row.time, row.target-row.focal, 0, color="C0",
                         head_width=0.2,length_includes_head=True)
        failed = prop[np.isnan(prop.target)]
        ax.scatter(failed.focal+0.2, failed.time,color="C0", marker='.')
    ax.set_ylim((tmax,0))
    ax.set(xlabel="Patch", ylabel='Time, $t$')
    ax.set(xticks=np.arange(D)+1, xlim=(0.5,D-1+0.5))

fig, ax = plt.subplots(1,2, figsize=(12,10))
plot_dyn(data_cl[-1], ax=ax[0])
plot_dyn(data_sm[-1], ax=ax[1])
ax[0].set(title=r"Main model")
ax[1].set(title=r"Smoothed model")
im = plt.imread('traj_legend.png')

newax = fig.add_axes([0.52, 0.70,  0.15, 0.15], anchor='NW')
newax.imshow(im)
newax.set(xticks=[],yticks=[], title='Legend')

data_cl[-1].to_csv("source_data/s8a_classic_trajectory.csv")
data_sm[-1].to_csv("source_data/s8b_smooth_trajectory.csv")
plt.savefig("fig/supfig/s8_size_select_traj.pdf", bbox_inches='tight')
plt.savefig("fig/supfig/s8_size_select_traj.svg", bbox_inches='tight')
```

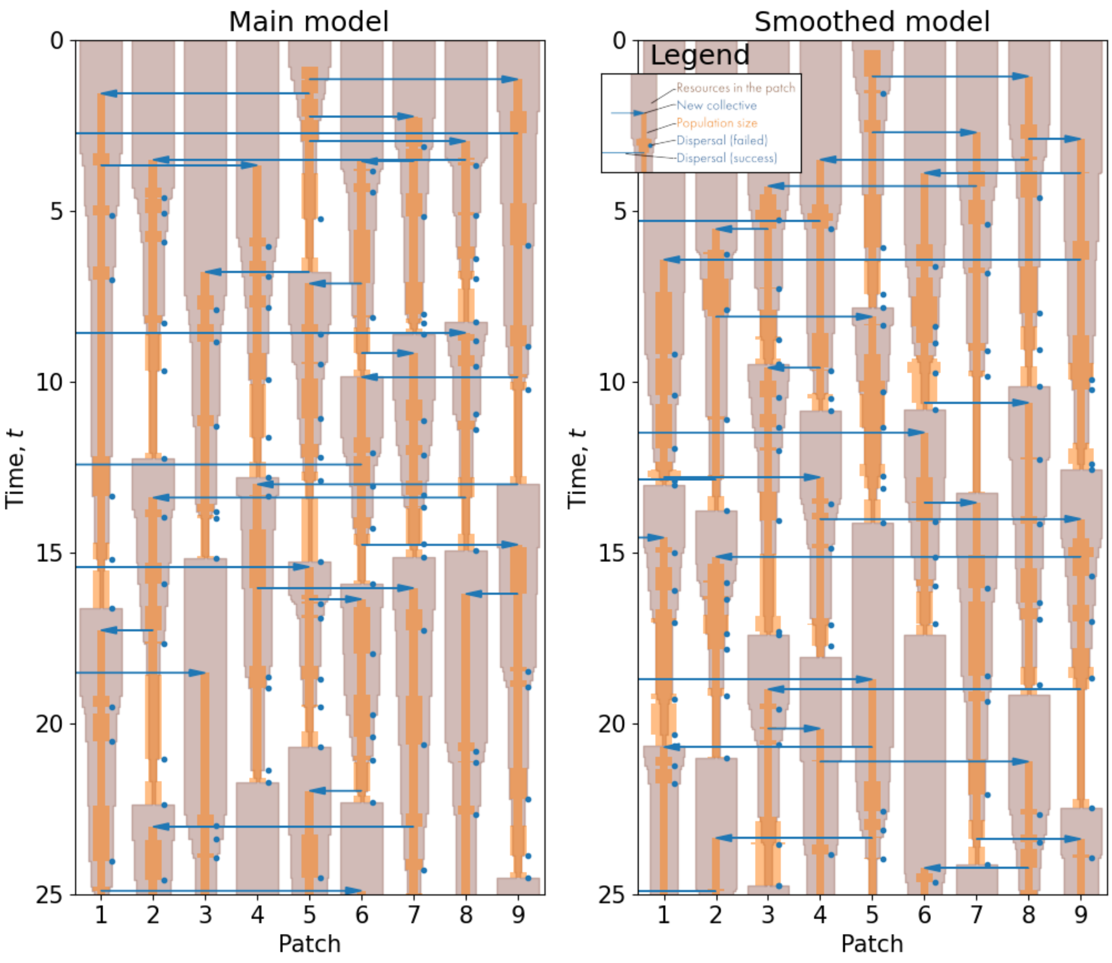
Main model
Smoothed model
Legend
Resources in the patch
New collective
Population size
Dispersal (failed)
Dispersal (success)
Time, t
0
5
10
15
20
25
Patch
1
2
3
4
5
6
7
8
9
