# Supplementary figures and images for: Stability of ecologically scaffolded traits during evolutionary transitions in individuality
Source: Nat Commun. 2024 Aug 3;15:6566. doi: 10.1038/s41467-024-50625-1 (PMC11297203; doi:10.1038/s41467-024-50625-1)

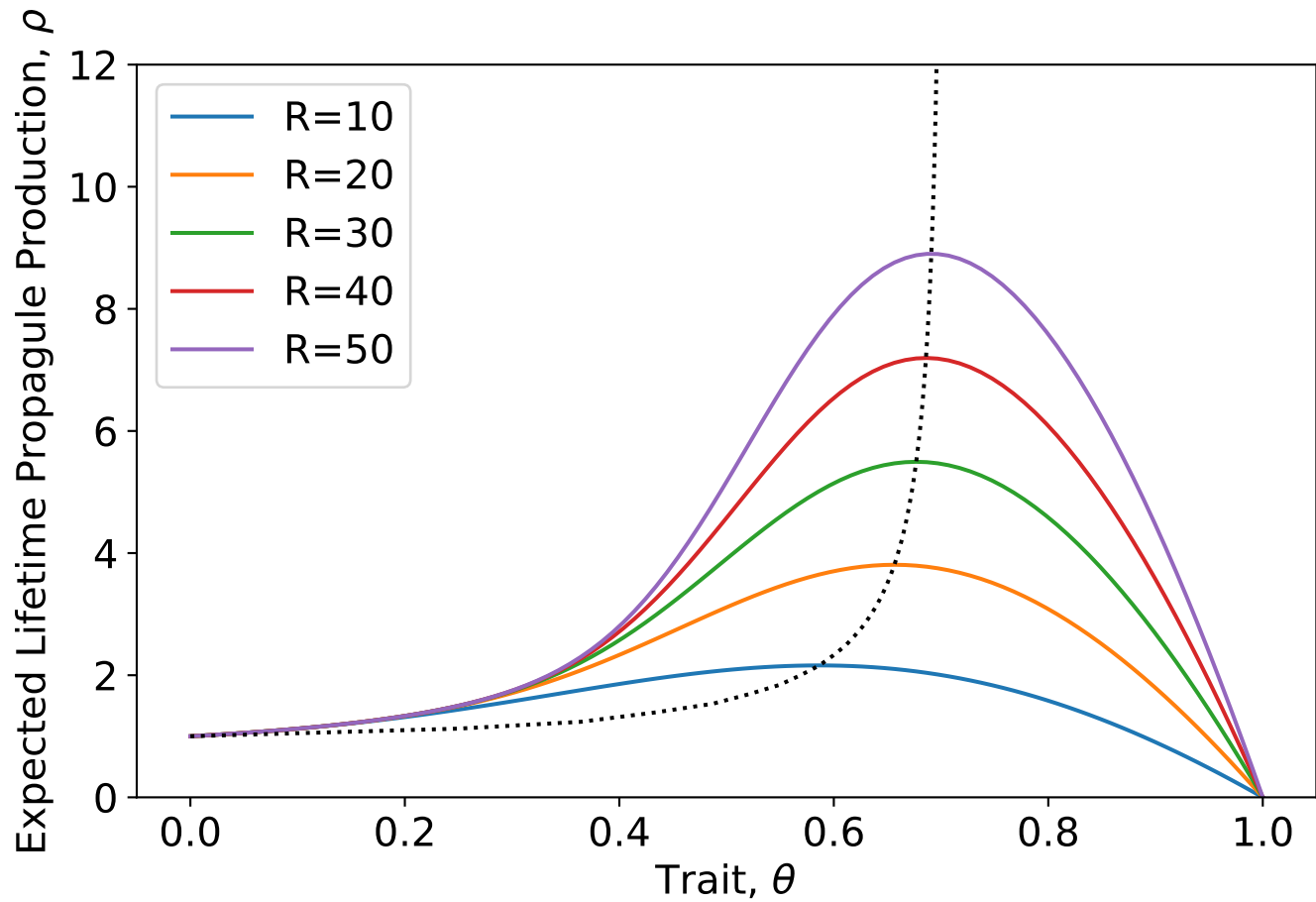

Supplement: Supplementary file 4 — Supplementary Code 1 [file 41467_2024_50625_MOESM4_ESM.zip › code/results/fig/2b_rho_pconstant.pdf]

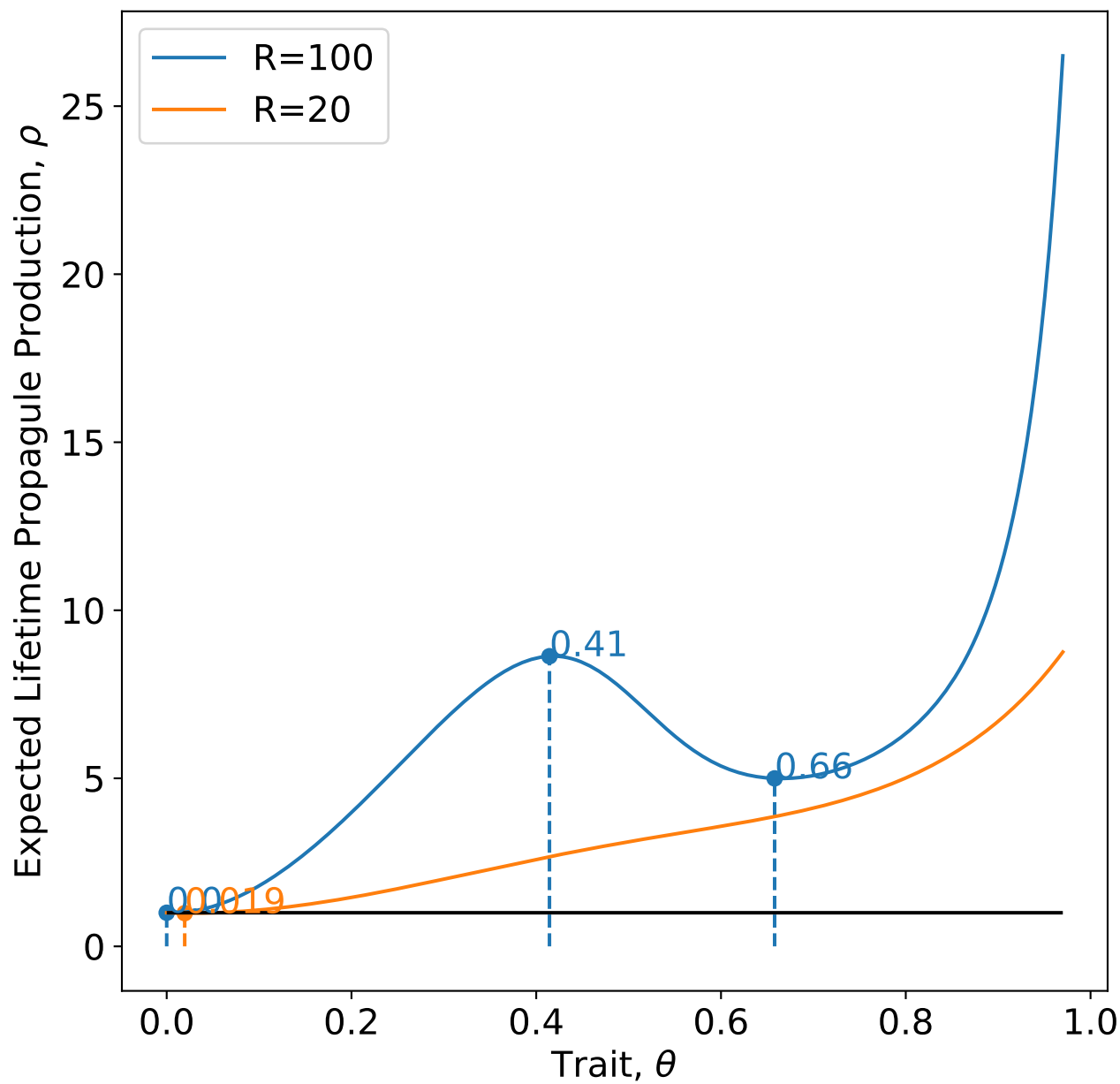

Supplement: Supplementary file 4 — Supplementary Code 1 [file 41467_2024_50625_MOESM4_ESM.zip › code/results/fig/4c_rho_function_of_theta_density_dep_model.pdf]

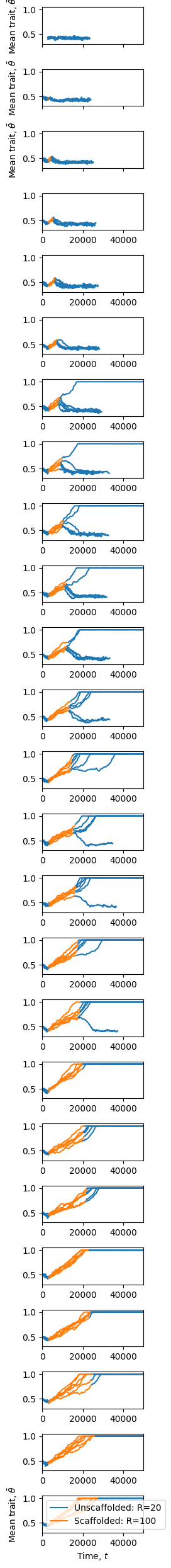

Supplement: Supplementary file 4 — Supplementary Code 1 [file 41467_2024_50625_MOESM4_ESM.zip › code/results/fig/4d_scaffold_trajectory_separated.png]

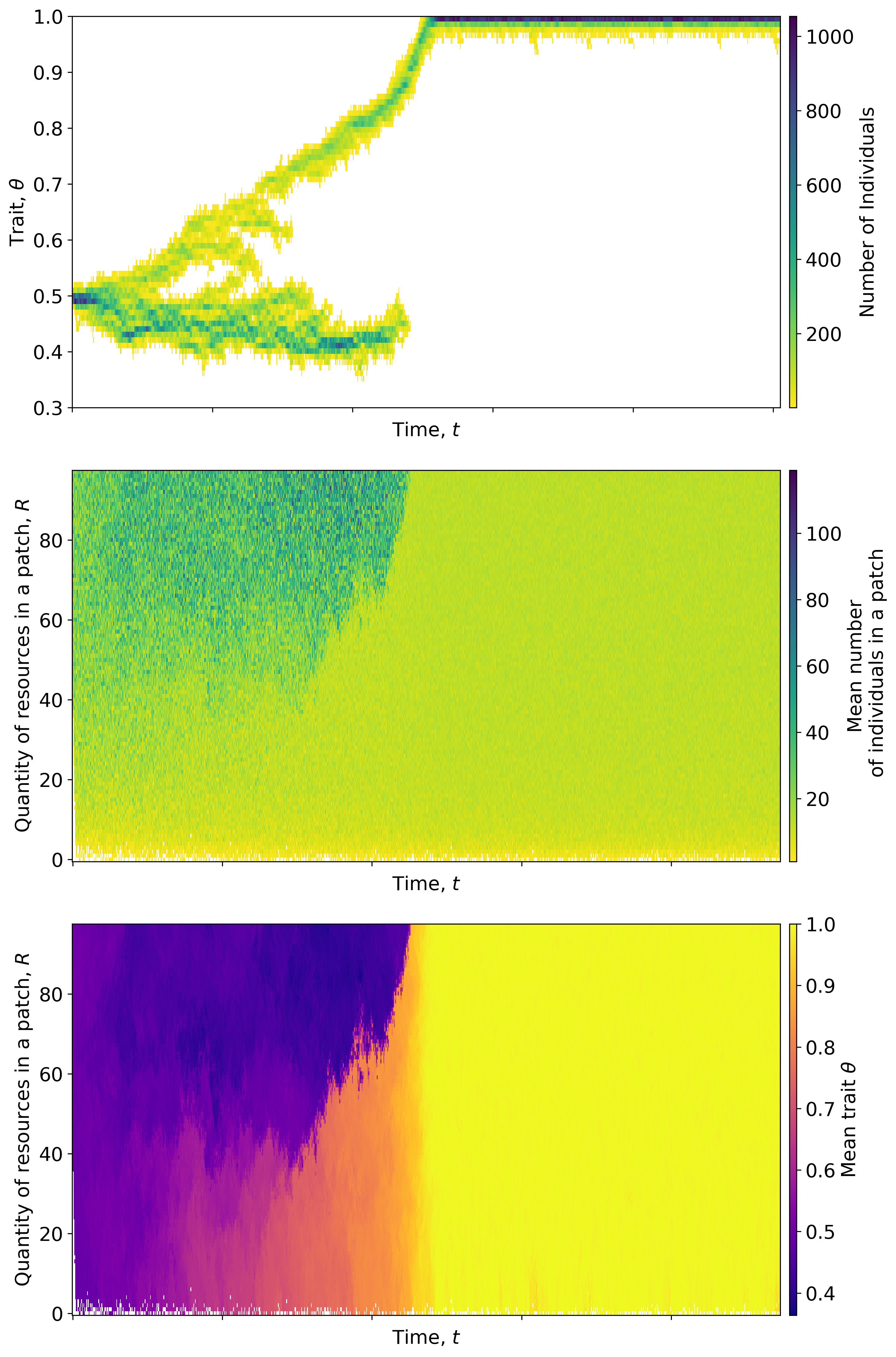

Supplement: Supplementary file 4 — Supplementary Code 1 [file 41467_2024_50625_MOESM4_ESM.zip › code/results/fig/5_gradient.png]

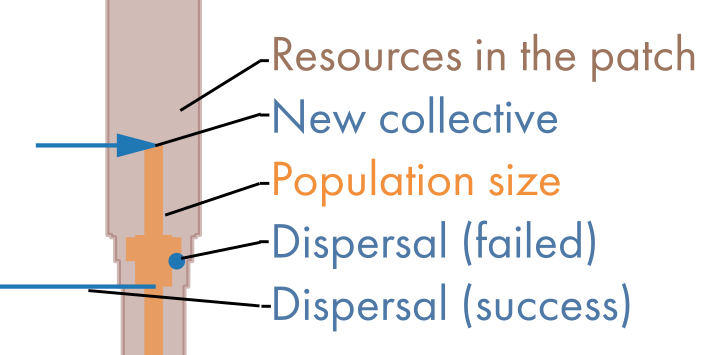

Supplement: Supplementary file 4 — Supplementary Code 1 [file 41467_2024_50625_MOESM4_ESM.zip › code/results/traj_legend.png]
